# Antisense transcription from stress-responsive transcription factors fine-tunes the cold response in Arabidopsis

**DOI:** 10.1093/plcell/koae160

**Published:** 2024-05-27

**Authors:** Shiv Kumar Meena, Marti Quevedo, Sarah Muniz Nardeli, Clément Verez, Susheel Sagar Bhat, Vasiliki Zacharaki, Peter Kindgren

**Affiliations:** Umeå Plant Science Centre, Department of Forest Genetics and Plant Physiology, Swedish University of Agricultural Sciences, Umeå 90187, Sweden; National Institute of Plant Genome Research, Aruna Asaf Ali Marg, New Delhi 110067, India; Umeå Plant Science Centre, Department of Plant Physiology, Umeå University, Umeå 90187, Sweden; Umeå Plant Science Centre, Department of Plant Physiology, Umeå University, Umeå 90187, Sweden; Umeå Plant Science Centre, Department of Forest Genetics and Plant Physiology, Swedish University of Agricultural Sciences, Umeå 90187, Sweden; Umeå Plant Science Centre, Department of Forest Genetics and Plant Physiology, Swedish University of Agricultural Sciences, Umeå 90187, Sweden; Umeå Plant Science Centre, Department of Forest Genetics and Plant Physiology, Swedish University of Agricultural Sciences, Umeå 90187, Sweden; Umeå Plant Science Centre, Department of Forest Genetics and Plant Physiology, Swedish University of Agricultural Sciences, Umeå 90187, Sweden

## Abstract

Transcription of antisense long noncoding RNAs (lncRNAs) occurs pervasively across eukaryotic genomes. Only a few antisense lncRNAs have been characterized and shown to control biological processes, albeit with idiosyncratic regulatory mechanisms. Thus, we largely lack knowledge about the general role of antisense transcription in eukaryotic organisms. Here, we characterized genes with antisense transcription initiating close to the poly(A) signal of genes (PAS genes) in Arabidopsis (*Arabidopsis thaliana*). We compared plant native elongation transcript sequencing (plaNET-seq) with RNA sequencing during short-term cold exposure and detected massive differences between the response in active transcription and steady-state levels of PAS gene-derived mRNAs. The cold-induced expression of transcription factors *B-BOX DOMAIN PROTEIN28* (*BBX28*) and *C2H2-TYPE ZINC FINGER FAMILY PROTEIN5* (*ZAT5*) was detected by plaNET-seq, while their steady-state level was only slightly altered due to high mRNA turnover. Knockdown of *BBX28* and *ZAT5* or of their respective antisense transcripts severely compromised plant freezing tolerance. Decreased antisense transcript expression levels resulted in a reduced cold response of *BBX28* and *ZAT5*, revealing a positive regulatory role of both antisense transcripts. This study expands the known repertoire of noncoding transcripts. It highlights that native transcription approaches can complement steady-state RNA techniques to identify biologically relevant players in stress responses.

## Teaser

Genes that host antisense transcripts are often stress-responsive and important for plant stress acclimation, even though their mRNA steady-state level is only marginally altered by stress.

## Introduction

Widespread long noncoding transcription from the complementary DNA (antisense) strand is present at thousands of protein-coding gene loci in eukaryotic organisms. Recent research in plants has evidenced antisense transcription for 30% of expressed genes in the model plant Arabidopsis (*Arabidopsis thaliana*) and for 60% of the genes in rice (Chen et al. 2019; Kindgren et al. 2019). Our current understanding is that the majority of antisense transcripts, also called *cis*-natural antisense transcripts (*cis*-NATs), are long noncoding transcripts [>200 nucleotides in length, long noncoding RNAs (lncRNAs)], but their general functional significance remains elusive (Reis and Poirier 2021). Complementarity between *cis*-NATs and the sense transcript, coupled with the overlapping spatiotemporal expression of sense–antisense pairs, endows them with the potential to engage in the formation of double-stranded RNA (dsRNA). dsRNA could undergo subsequent detection by the factors of RNA silencing machinery such as Dicer or Dicer-like (DCL) and Argonaute (AGO) family proteins. However, there is weak evidence that endogenous small interfering RNAs are derived from NAT-sense pairs (Henz et al. 2007; Reis and Poirier 2021). Only a handful of studies from different plant species describe a negative role of NATs over the sense transcription (Borsani et al. 2005; Katiyar-Agarwal et al. 2006; Swiezewski et al. 2007; Held et al. 2008; Wan et al. 2016). So far, few *cis*-NATs in plants have been experimentally characterized with mechanistic insights, thereby limiting our understanding of their general function (Wierzbicki et al. 2021). Thus, we only have a rudimentary knowledge of the roles of antisense transcription, and its widespread prevalence is certainly an enigma in modern plant research.

Our global numerical comprehension of noncoding transcription has been mostly based on sequencing technologies that fundamentally use the steady-state RNA detection principle. For example, in Arabidopsis, 70% of mRNAs were postulated to form sense–antisense pairs based on RNA-sequencing (RNA-seq) experiments (Wang et al. 2014). However, noncoding transcription is far more pervasive and complex than visualized by canonical steady-state analyses. A major challenge in the field is the detection of antisense and other noncoding transcription due to the low abundance and high turnover rate (Mayer et al. 2015; Kindgren et al. 2019; Thieffry et al. 2020). Classical steady-state level detection methods, such as RNA-seq, are ill-suited to investigate noncoding transcription. In contrast, a technique that investigates active transcription (i.e. native elongation transcript sequencing [NET-seq]) is better suited for detecting rapidly degraded RNA species (Mayer et al. 2015). NET-seq in plants (plaNET-seq or pNET-seq) captures actively transcribing RNA Polymerase II (RNAPII) complexes and enables strand-specific sequencing of the RNA molecules associated with the captured RNAPII complexes before any degradation can occur, thereby uncovering all transcription events genome-wide (Zhu et al. 2018; Kindgren et al. 2019). Case-in-point, plaNET-seq detected ∼8,000 unannotated long noncoding transcripts, many of them being antisense transcripts initiating from the host gene's 3′-end (poly(A) antisense genes, PAS genes) (Kindgren et al. 2019).

Emerging evidence indicates that antisense transcription is essential for plant development and stress responses (Matsui et al. 2008; Thieffry et al. 2020). However, the underlying molecular mechanism(s) involved in sense/antisense transcriptional crosstalk often seem idiosyncratic with few common components involved (reviewed in Lucero et al. 2021). Nevertheless, antisense RNAs have been characterized to control seed germination (Fedak et al. 2016), phosphate starvation (Jabnoune et al. 2013), flowering control (Swiezewski et al. 2007; Henriques et al. 2017), hormonal regulation (Ariel et al. 2020), and cold acclimation (Kindgren et al. 2018). Cold acclimation, initiated by transcriptional processes, allows plants to adapt and eventually withstand freezing. At present, cold stress is the sole abiotic stress monitored by plaNET-seq (Kindgren et al. 2019). Remarkably, in plaNET-seq datasets, antisense transcription at PAS genes responds rapidly to cold with a global downregulation, suggesting a putative role in the cold response (Kindgren et al. 2019). Thus, exposure to cold temperature is an excellent environmental cue for studying antisense transcription's role in Arabidopsis.

In this study, we use plaNET-seq and RNA-seq data to show an earlier hidden layer of genes involved in cold acclimation. We show that RNA-seq data poorly captures the cold stress-induced transcriptional changes of protein-coding genes. On the contrary, plaNET-seq efficiently uncovers these transcriptional changes since transcription is captured prior to their degradation. Among those, we identified 2 cold-responsive transcription factors (TFs) that responded natively as crucial for cold acclimation in Arabidopsis. Importantly, experimental validation demonstrated that their antisense transcription is positively correlated to the stress-responsiveness of the sense transcription, suggesting a role for antisense transcription in assisting certain TFs’ stress responses, such as cold exposure.

## Results

### PAS genes are enriched in stress-responsive TFs with high active transcription

**Figure 1.**
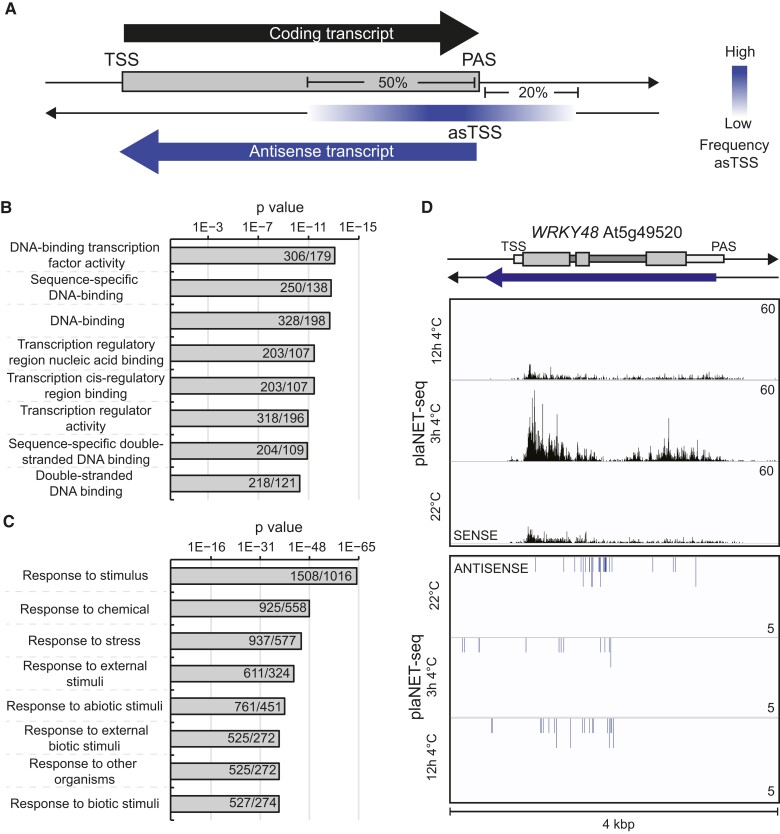
PAS genes are overrepresented by stress-responsive TFs. **A)** Graphical representation of the definition of PAS genes. TSS, transcription start site; asTSS, antisense TSS. **B)** GO term enrichment of PAS genes (molecular function). The number in bars indicates the found number of genes/expected number of genes. **C)** GO term enrichment of PAS genes (biological processes). The number in bars indicates the found number of genes/expected number of found genes. **D)** Example of a PAS gene (*WRKY48*, At5g49520). Screenshots showing plaNET-seq expression profile from datasets at 22° C, 3 h, and 12 h post cold treatment. Elevated transcriptional activity is indicated by higher peak density and amplitude.

Genes that host PAS transcription (PAS genes) represent over 3,000 genes in Arabidopsis and were recently detected by plaNET-seq (Kindgren et al. 2019). PAS genes are defined as protein-coding genes with antisense transcription initiating in the 3′-half or 20% of the gene length downstream of the sense gene's PAS (Fig. 1A and Supplementary Data Set 1). Characterization of PAS genes has not been done rigorously in Arabidopsis, so we first investigated which biological processes and molecular functions were overrepresented in the list of all PAS genes (Fig. 1, B and C and Supplementary Data Set 2). Interestingly, there was a clear enrichment of stress-responsive genes that are involved in DNA binding [in particular transcription factors (TFs)]. Therefore, for our further analysis, we decided to divide the PAS genes into PAS TFs (*n* = 294) and PAS non-TFs. We also included TFs without PAS (TFs non-PAS) as a control group. An example of a known stress-responsive gene encoding a TF with an antisense transcript that responds natively to cold temperature was *WRKY48* (Fig. 1D; Xing et al. 2008).

**Figure 2.**
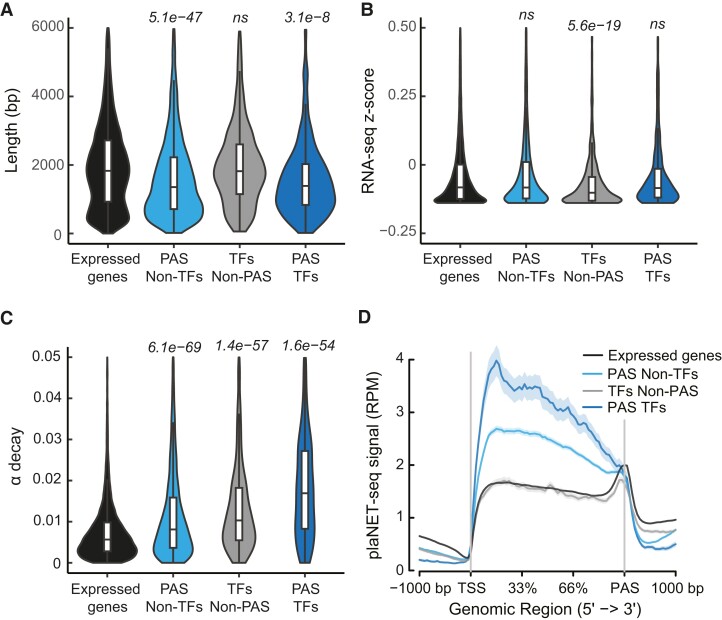
PAS genes are highly expressed, but their mRNA is degraded rapidly. **A)** Violin plot of the length of PAS non-TF genes, TFs non-PAS, PAS TF genes, and all expressed genes. Centerline, median; box limits, upper and lower quartiles; whiskers, 1.5× interquartile range. *P-*value was calculated with Mann–Whitney *U* test and *P* < 0.05 was considered significant. **B)** Violin plot of the steady-state level of PAS non-TF genes, TFs non-PAS, PAS TF genes, and all expressed genes. Centerline, median; box limits, upper and lower quartiles; whiskers, 1.5× interquartile range. *P*-value was calculated with Mann–Whitney *U* test and *P* < 0.05 was considered significant. **C)** Violin plot of the decay rate of PAS non-TF genes, TFs non-PAS, PAS TF genes, and all expressed genes after transcriptional inhibition by cordycepin. Centerline, median; box limits, upper and lower quartiles; whiskers, 1.5× interquartile range. *P*-value was calculated with Mann–Whitney *U* test and *P* < 0.05 was considered significant. **D)** Metagene analysis of plaNET-seq data of PAS non-TF genes, TFs non-PAS, PAS TF genes, and all expressed genes. The shaded area shows a 95% confidence interval for the mean.

Overall, PAS genes tended to be shorter than other expressed genes and TFs non-PAS (Fig. 2A) and the steady-state levels of PAS non-TFs or PAS TFs gene mRNA were not significantly different from those of all expressed genes (Fig. 2B). Surprisingly, PAS gene mRNA had a greater turnover rate compared with all expressed genes when measured by RNA-seq after treatment with the transcription inhibitor, cordycepin (alpha decay) (Fig. 2C) (Sorenson et al. 2018). TFs non-PAS also showed a significantly higher turnover rate, suggesting that the rapid degradation of mRNA in these classes of genes is independent of antisense transcription. We confirmed these results with data from transcription inhibition with another inhibitor, actinomycin D (Supplementary Fig. S1A) (Narsai et al. 2007). Both PAS non-TFs and PAS TFs genes displayed high active RNAPII transcription at the genome-wide level compared with expressed genes and TFs non-PAS (Fig. 2D and Supplementary Fig. S1B). In addition, PAS TFs showed a higher RNAPII occupancy compared with PAS non-TFs (Supplementary Fig. S1B). Thus, the higher expression level of PAS genes and high turnover rate explained the small effects on RNA steady-state levels. Taken together, PAS genes are enriched in stress-responsive TFs with high transcriptional and post-transcriptional regulation. Moreover, the high turnover rate seems to be a general feature of TF mRNAs and not a consequence of PAS TF antisense transcription.

### The expression levels of cold-responsive genes detected by plaNET-seq correlate poorly with RNA-seq

**Figure 3.**
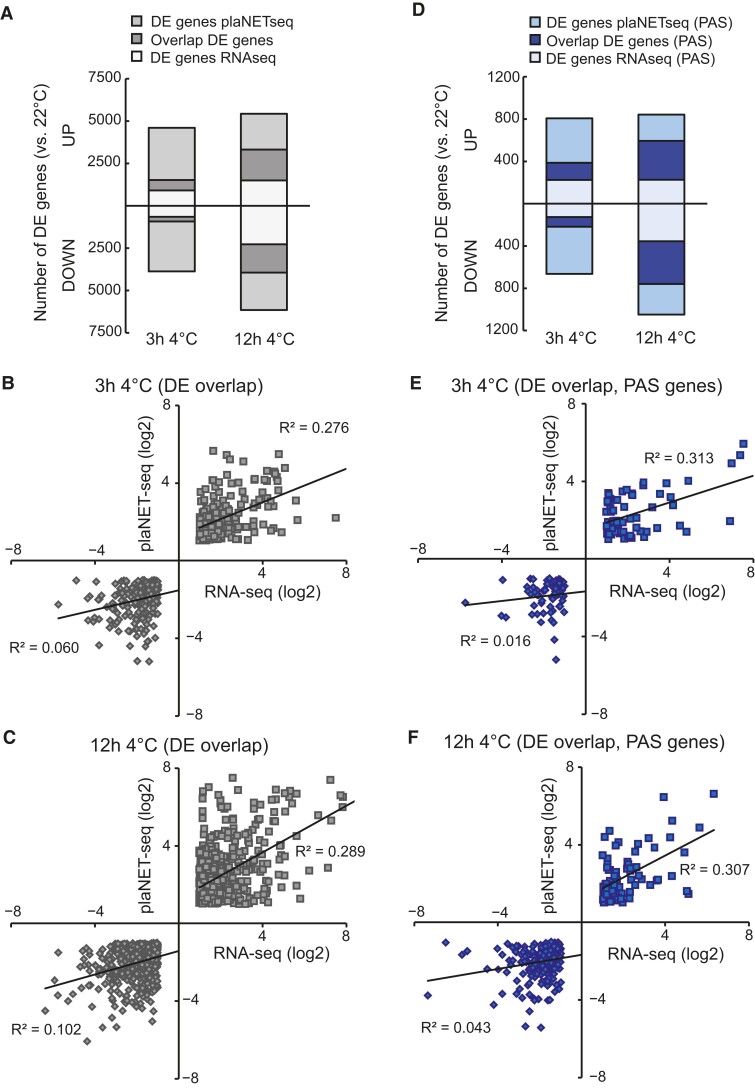
Discrepancy between plaNET-seq and RNA-Seq. **A)** Number of total genes differentially expressed in RNA-seq and plaNET-seq after 3 and 12 h of 4 °C exposure. The overlap between the 2 sequencing techniques is shown in darker gray. **B)** and **C)** Correlation plots between differentially expressed genes in RNA-seq and plaNET-seq (UP: squares, DOWN: diamonds) after **B)** 3 h of 4 °C and **C)** 12 h of 4 °C. The genes plotted come from the overlap seen in **A)**. **D)** Number of PAS genes differentially expressed in RNA-seq and plaNET-seq after 3 and 12 h of 4 °C exposure. The overlap between the 2 sequencing techniques is shown in blue. **E)** and **F)** Correlation plots between differentially expressed PAS genes in RNA-seq and plaNET-seq (UP: squares, DOWN: diamonds) after **E)** 3 h of 4 °C and **F)** 12 h of 4 °C. The genes plotted come from the overlap seen in **D)**.

The high transcription activity of PAS genes and rapid turnover of their mRNA made us interested in comparing the genome-wide steady-state levels of mRNA to how genes are actively transcribed (plaNET-seq). Thus, we performed RNA-seq using samples from seedlings grown similarly to the plaNET-seq experiment (10 d in long-day conditions at 22 °C and cold stress samples from 3 h at 4 °C and 12 h at 4 °C). In our cold-treated RNA-seq dataset, many mRNAs drastically changed their steady-state level (Fig. 3A and Supplementary Data Set 3). When we compared differentially expressed (DE) genes (fold change) between our RNA-seq and plaNET-seq datasets, we saw that RNA-seq poorly reflected the genes differentially expressed determined by plaNET-seq (Kindgren et al. 2019). The overlap was only 616 of the upregulated (UP) genes and 270 of the downregulated (DOWN) genes in the same direction after 3 h at 4 °C (Fig. 3A). After 12 h at 4 °C, we observed increased overlap, 1,819 of UP genes and 1,674 of DOWN genes (Fig. 3A). In addition, we saw a low correlation between the DE gene fold change at both time points (Fig. 3, B and C). This suggests that there is a clear discrepancy between the active transcriptional changes (plaNET-seq) and changes to the steady-state levels of mRNA (RNA-seq) occurring during cold stress, especially early in the cold response.

**Figure 4.**
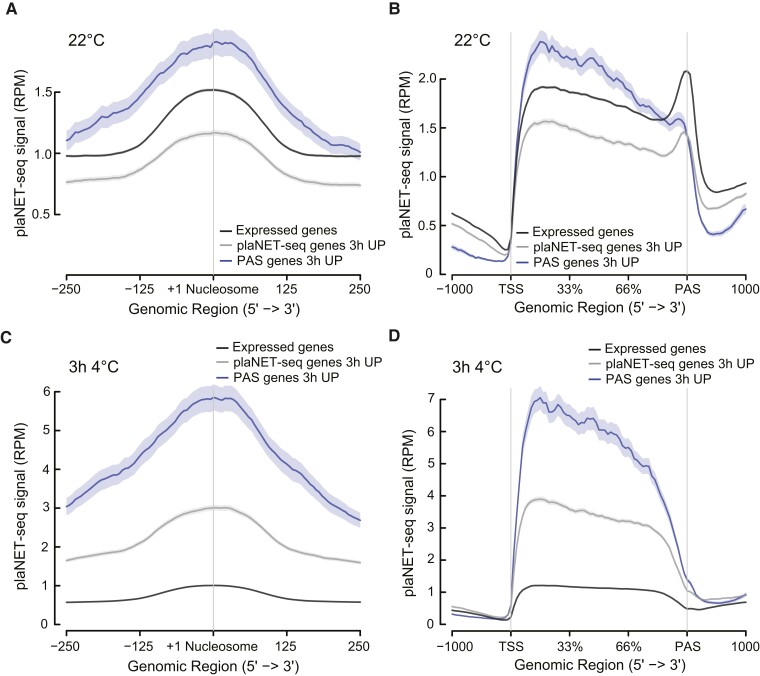
PAS genes have enhanced cold-responsiveness. **A)** Metagene analysis of the plaNET-seq signal (at 22 °C) in a 500 bp window centered at the +1 nucleosome. PAS genes that will be UP after 3 h at 4 °C, genes without antisense transcription but UP after 3 h at 4 °C, and non-DE genes are shown. The shaded area shows a 95% confidence interval for the mean. **B)** Metagene analysis of plaNET-seq data (at 22 °C). PAS genes that will be UP after 3 h at 4 °C, genes without antisense transcription but UP after 3 h at 4 °C, and non-DE genes are shown. The shaded area shows a 95% confidence interval for the mean. **C)** Metagene analysis of the plaNET-seq signal (after 3 h at 4 °C) in a 500 bp window centered at the +1 nucleosome. PAS UP genes after 3 h at 4 °C, genes without antisense transcription but UP after 3 h at 4 °C, and non-DE genes are shown. The shaded area shows a 95% confidence interval for the mean. **D)** Metagene analysis of plaNET-seq data (after 3 h at 4 °C). PAS UP genes after 3 h at 4 °C, genes without antisense transcription but UP after 3 h at 4 °C, and non-DE genes are shown. The shaded area shows a 95% confidence interval for the mean.

Focusing on the PAS genes, we saw a similar pattern as in all DE genes (Fig. 3, D to F). Astoundingly, we found that 2,214 (71%) PAS genes changed their expression significantly to cold (either in plaNET-seq or RNA-seq data) at one or both cold time points, corroborating their responsiveness to stress (Supplementary Data Set 4). Out of these genes, 229 were PAS TFs (78% were responsive to cold, Supplementary Data Set 4). To further confirm that PAS genes are highly responsive to cold, we compared PAS genes UP after 3 h 4 °C with UP genes without antisense transcription (determined by plaNET-seq). At 22 °C, PAS genes showed an increased RNAPII stalling around the +1 nucleosome, a hallmark for stress-responsiveness compared with other UP and non-DE genes (Fig. 4A) and an overall high active transcription (Fig. 4B). After 3 h 4 °C, PAS UP genes showed a more extreme response to cold temperature compared with other UP genes (Fig. 4, C and D), suggesting that, indeed, PAS genes have an enhanced responsiveness to cold compared with other genes. In contrast, antisense transcription for UP PAS genes remained similar throughout the cold response (Supplementary Fig. S2). Taken together, these data strengthen the hypothesis that PAS genes are inclined to respond to cold temperatures, but there is no correlation between PAS-host gene expression and antisense expression during the initial cold response. In addition, the discrepancy between plaNET-seq and RNA-seq argues that some TFs might have been overlooked as cold-responsive genes and highlight the use of combining nascent RNA methods with steady-state levels of mRNA to fully understand the cold response in an organism.

### The discrepancy between active transcription and steady-state levels can partly be explained by mRNA turnover rates

**Figure 5.**
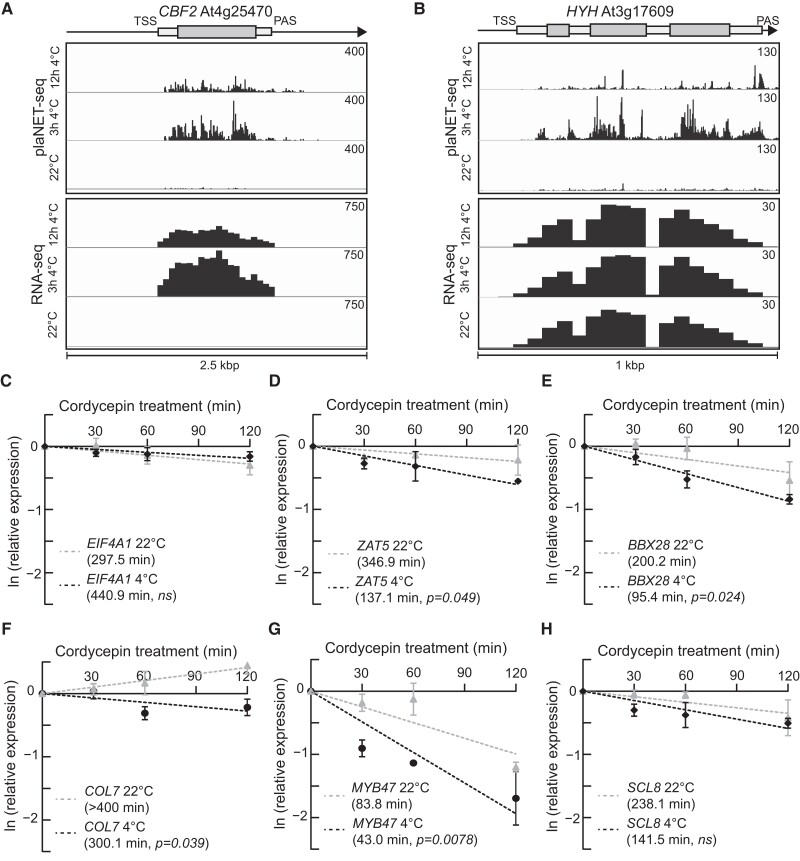
*ZAT5* and *BBX28* mRNA show decreased stability at 4 °C. **A)** At4g254970 (*CBF2*). Screenshots are from plaNET-seq and RNA-seq datasets. Elevated transcriptional activity is indicated by higher peak density and amplitude. **B)** At3g17609 (*HYH*). Screenshots are from plaNET-seq and RNA-seq datasets. Elevated transcriptional activity is indicated by higher peak density and amplitude. **C)** to **H)** Transcript stability assays for **C)***EIF4A1*, **D)***ZAT5*, **E)***BBX28*, **F)***COL7*, **G)***MYB47*, and **H)***SCL8* after transcriptional inhibition with cordycepin at 22 °C and 4 °C. Half-life (*t*_1/2_) was determined from the slope of degradation curves that were obtained after RT-qPCR analysis of cordycepin-treated seedlings at indicated time points. Each data point is the mean of 3 biological replicates. Error bars represent ± SD.

Next, we focused on the differentially expressed TFs with antisense transcription. We aimed to identify additional biologically important TFs in the cold response. A special emphasis was put on the expression pattern in plaNET-seq as the distinct dynamics of PAS TFs mRNAs (Fig. 2) might have masked cold-responsive TFs in earlier studies. We reasoned that rapidly responsive TFs had a high probability of being involved in cold acclimation since this expression pattern mirrors that of known assigned TFs in the cold response, such as C-REPEAT BINDING FACTOR2 (CBF2, At4g25470) (Fig. 5A). *CBF2* is massively induced and transiently peaks after 3 h at 4 °C. Out of the 242 PAS TFs that responded to cold, 25 showed a similar plaNET-seq expression pattern to *CBF2* (UP after 3 h and DOWN between 3 and 12 h) (Supplementary Data Set 5). Only 9 of the 25 genes showed upregulation by RNA-seq after 3 h (Supplementary Data Set 5). An example of a gene that was DE in plaNET-seq but not in RNA-seq was *HY5-HOMOLOG* (*HYH*, At3g17609) (Fig. 5B).

Due to the RNA degradation characteristics of PAS TFs, we first tested the mRNA turnover rates (by cordycepin incubation) for 5 randomly chosen genes from the 25 genes with convincing expression patterns in plaNET-seq and no or slight upregulation by RNA-seq. MYB DOMAIN PROTEIN-47 (MYB47, At1g18710) has a potential role in drought and hormone signaling (Ding et al. 2014; Marquis et al. 2022). CONSTANS-LIKE 7 (COL7, At1g73870) is involved in the shade avoidance response (Wang et al. 2013). The C2H2-type zinc finger family proteins, *C2H2-TYPE ZINC FINGER FAMILY PROTEIN-5* (ZAT5, At2g28200) and SCARECROW-LIKE-8 (SCL8, At5g52510), are uncharacterized proteins and B-BOX DOMAIN PROTEIN-28 (BBX28, At4g27310) has been characterized for its involvement in flowering and photomorphogenesis (Song et al. 2020; Cao et al. 2022). *MYB47*, *SCL8*, and *BBX28* showed a slight upregulation in the RNA-seq data. However, neither BBX28, SCL8, MYB47, or any of the other chosen proteins have a known function in the cold response of Arabidopsis. Our assay control *EUKARYOTIC TRANSLATION INITIATION FACTOR-4A1* (*EIF4A1*), a stable mRNA at 22 °C, showed similar stability at 4 °C as compared with 22 °C (Fig. 5C). In addition, a recent study showed increased stability in cold for a key TF in the cold response, *CBF1* (Zacharaki et al. 2023). In contrast, all the PAS TF candidate genes’ mRNAs, except *SCL8*, showed a significantly decreased transcript stability at 4 °C compared with 22 °C (Fig. 5, D to H). Thus, induced transcriptional activity at gene loci responding to cold may remain partly undetected by steady-state methods due to rapid mRNA decay at 4 °C. Again, our results highlight that nascent transcription methods can complement RNA steady-state level methods to identify stress-responsive genes, in particular those with highly dynamic regulation and mRNA turnover.

### ZAT5 and BBX28 are involved in cold acclimation

**Figure 6.**
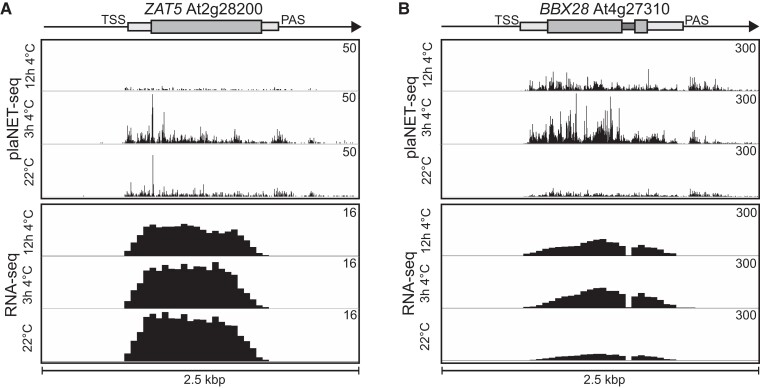
Examples of cold-responsive PAS genes. **A)***AT2G28200* (*ZAT5*). Screenshots are from plaNET-seq and RNA-seq datasets. Elevated transcriptional activity is indicated by higher peak density and amplitude. **B)***AT4G27310* (*BBX28*). Screenshots are from plaNET-seq and RNA-seq datasets. Elevated transcriptional activity is indicated by higher peak density and amplitude.

**Figure 7.**
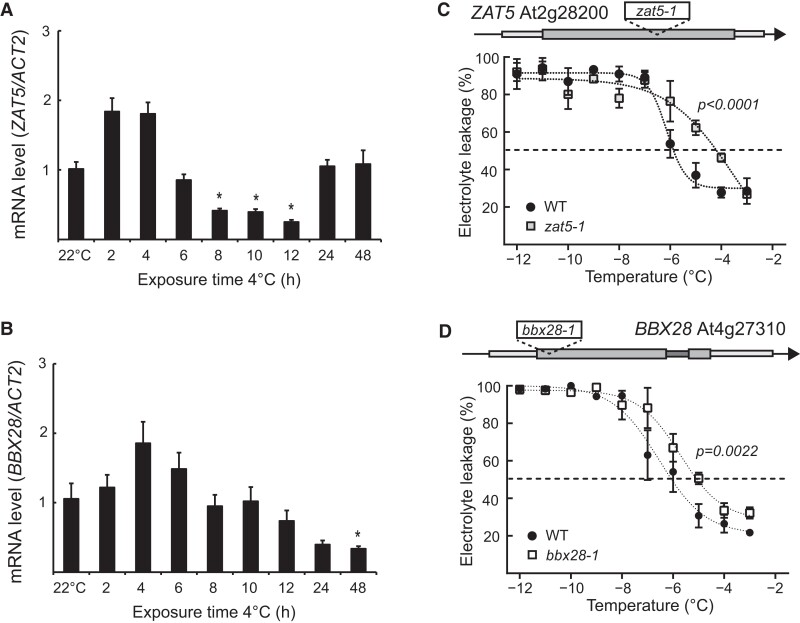
*ZAT5* and *BBX28* are important for cold acclimation. **A)** The relative steady-state level of *ZAT5* measured by RT-qPCR following cold exposure (4 °C). Steady-state levels were normalized to WT levels at 22 °C. The mean values are from 3 biological replicates. Error bars represent ± SEM. Statistical significance was calculated with Student's *t*-test (*P < 0.05). **B)** The relative steady-state level of *BBX28* measured by RT-qPCR following cold exposure (4 °C). Steady-state levels were normalized to WT levels at 22 °C. The mean values are from 3 biological replicates. Error bars represent ± SEM. Statistical significance was calculated with Student's *t*-test (*P < 0.05). **C)** Electrolyte leakage in WT and *zat5-1* of cold-acclimated (4 d of 4 °C) plants. Each data point represents the mean from at least 3 biological replicates (±SEM). The dashed line represents the threshold value, 50%. The dotted lines represent the curve fit. Statistical significance was calculated with an extra sum-of-squares *F*-test and the *P*-value is shown in the figure. **D)** Electrolyte leakage in WT and *bbx28-1* of cold-acclimated (4 d of 4 °C) plants. Each data point represents the mean from at least 3 biological replicates (±SEM). The dashed line represents the threshold value, 50%. The dotted lines represent the curve fit. Statistical significance was calculated with an extra sum-of-squares *F*-test and the *P-*value is shown in the figure.

Can mRNAs with minor differences in steady-state levels during stress have an important biological role in the stress adaptation for the plant? To answer this, we focused on 2 candidate genes for our continued analysis, *ZAT5* and *BBX28*. Both genes responded rapidly to cold temperatures, as detected by plaNET-seq (Fig. 6, A and B). We could not detect any significantly increased steady-state level of *ZAT5* after exposure to 4 °C with RT-qPCR, although a positive trend was observed early in the cold response (Fig. 7A). We did see a significant downregulation from control levels starting at 8 h of 4 °C (Fig. 7A). For *BBX28*, we could not detect any significant upregulation or downregulation, but a positive trend early in the cold response was observed (Fig. 7B). The discrepancy between our RT-qPCR and RNA-seq results may suggest that the upregulation of *BBX28* and *ZAT5* is slight and not always consistently statistically significant depending on separate cold treatments and the variation between replicates. However, since both *BBX28* and *ZAT5* have been found to be diurnally expressed (Romanowski et al. 2020), we also checked their steady-state levels with controls (seedlings kept at 22 °C) taken at the same time points as the cold samples (3 h and 12 at 4 °C). Here, we did see a small but significant increase of both *BBX28* and *ZAT5* after 3 h (Supplementary Fig. S3, A and B).

To test their biological importance in the cold acclimation process, we isolated 2 independent T-DNA lines disrupting the 2 genes and subjected these lines to a freezing test together with wild type (WT; Fig. 7, C and D). In the freezing test, leaf discs of non-acclimated and cold-acclimated plants (4 d at 4 °C) are in contact with water and exposed to decreasing freezing temperatures and measured for plasma membrane disruption (i.e. leakage of electrolytes). Therefore, measuring electrolyte leakage is a measurement of how well the cells can survive freezing temperatures. Indeed, we found that both mutant lines were impaired in their acclimation to cold. For *zat5-1*, the freezing tolerance was significantly lower (−3.7 ± 1.7 °C, *P <* 0.0001) compared with −6.1 ± 0.2 °C for WT. The freezing tolerance for *bbx28-1* was also significantly lower (−5.7 ± 0.2 °C, *P =* 0.0022) compared with −6.5 ± 0.2 °C for WT. We could not detect any difference in non-acclimated plants (Supplementary Fig. S3, C and D), suggesting that both *ZAT5* and *BBX28* have a specific role in the cold acclimation process in Arabidopsis. Overall, these results indicate that changes to the transcription activity with minor changes to the mRNA steady-state levels can have a significant biological role.

### Antisense transcription is required for proper regulation of ZAT5 and BBX28

**Figure 8.**
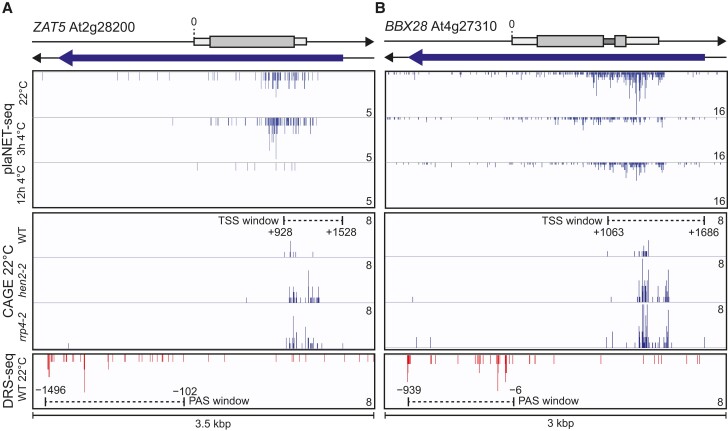
Characterization of *asZAT5* and *asBBX28*. Screenshots from plaNET-seq (upper panel), CAGE (middle panel), and DRS-seq (lower panel) for **A)***ZAT5* and **B)***BBX28*. 0 indicates the TSS of the sense transcript. Elevated transcriptional activity is indicated by higher peak density and amplitude.

Next, we turned to our second question; what is the role of antisense transcription along the *ZAT5* and *BBX28* gene body? Our plaNET-seq data revealed that both *ZAT5* and *BBX28* had antisense transcription affected by cold temperature, although the differences were small compared with sense expression (Fig. 8, A and B, upper panel). To identify the 5´ and 3′-end of *asZAT5* and *asBBX28*, we used available Cap Analysis of Gene Expression sequencing (CAGE) (Thieffry et al. 2020) and Direct RNA Sequencing (DRS) data (Schurch et al. 2014) (Fig. 8, A and B, middle and lower panels). Interestingly, both antisense transcripts are targets of the nuclear exosome (see CAGE data for the exosome mutants *hen2-2* and *rrp4-2*). Consequently, 5′-ends are more prominent in the exosome mutants, suggesting that the transcripts are degraded rapidly after their synthesis. We could see that there was no precise start or end to the antisense transcription, but rather a window at both ends. Antisense transcription starts well beyond the poly(A)-site of their host genes and navigates until at least 1 kb upstream of the host gene's transcription start site (TSS).

To investigate the role of the antisense transcription at the translational level, firefly luciferase (*LUC*) reporter constructs for *ZAT5* were generated, using GreenGate cloning system (Lampropoulos et al. 2013), with and without the 1,403 bp DNA sequence that harbors *ZAT5* 3′ UTR, associated PAS antisense transcript and putative promoter, i.e. *ProZAT5:ZAT5-LUC-UTR-ASProZAT5* and *ProZAT5:ZAT5-LUC-tNOS*. The *Agrobacterium tumefaciens* cultures carrying these plasmids were used for infiltration of *Nicotiana benthamiana* leaves for transient expression assay (Supplementary Fig. S4, A and B). The first construct included the endogenous promoter and cDNA fused to the *LUC* gene. Downstream of the *LUC* gene was the endogenous untranslated 5′-region of *ZAT5* and the antisense (AS) promoter. In the second construct, the *ZAT5* promoter and cDNA were fused to LUC followed by the strong tNOS terminator. The tNOS terminator diminishes any antisense transcription over the *LUC* and *ZAT5* gene body (Kindgren et al. 2018). The endogenous construct (construct 1 in Supplementary Fig. S4) showed an induction after cold exposure, confirming that the *ZAT5* promoter is cold-responsive and corresponds with positive transcriptional regulation of *ZAT5* by its antisense (Supplementary Fig. S4C). At both 22 °C and 4 °C, the tNOS construct showed lower LUC activity compared with the full-length construct (Supplementary Fig. S4, D and E). The overall cold-responsiveness decreased from 2.5-fold in the full-length construct to 2.0-fold in the tNOS construct, suggesting that the antisense transcription over *ZAT5* has a positive role in the transcription level and stress-responsiveness of the gene.

**Figure 9.**
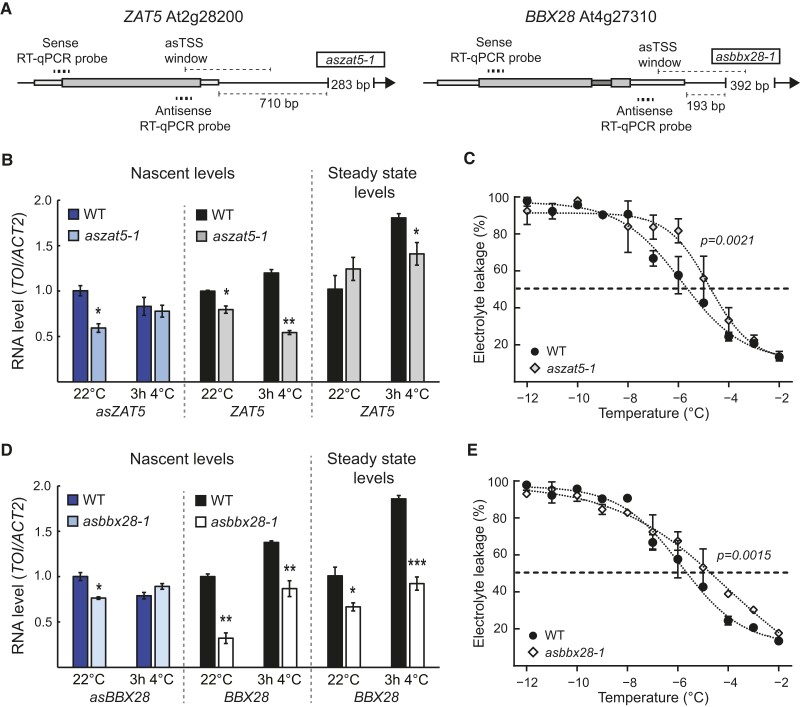
*asZAT5* and *asBBX28* are important for the proper regulation of their host gene. **A)** Graphical representation of the *ZAT5* and the *BBX28* loci showing the location of sequence targeted by CRISPR-Cas9 to generate an *asZAT5* knockdown line (*aszat5-1*) and an *asBBX28* knockdown line (*asbbx28-1*). Antisense transcription start site (asTSS) window and distances of knocked out the genomic sequence from 3′-end are marked with dotted lines. The location of RT-qPCR probes for sense and antisense are shown. **B)** The relative nascent and steady-state level of *asZAT5* and *ZAT5* in WT and *aszat5-1* measured by RT-qPCR at 22 °C and following 3 h of cold exposure (4 °C). All levels were normalized to WT levels at 22 °C. The mean values are from 3 biological replicates. Error bars represent ± SEM. Statistical significance was calculated with Student's *t*-test (*P < 0.05). **C)** Electrolyte leakage in WT and *aszat5-1* of cold-acclimated (5 d of 4 °C) plants. Each data point represents the mean from at least 3 biological replicates (±SEM). The dashed line represents the threshold value, 50%. The dotted lines represent the curve fit. Statistical significance was calculated with an extra sum-of-squares *F*-test and the *P-*value is shown in the figure. **D)** The relative steady-state level of *asBBX28* and *BBX28* in WT and *asbbx28-1* measured by RT-qPCR at 22 °C and following 3 h of cold exposure (4 °C). All levels were normalized to WT levels at 22 °C. The mean values are from 3 biological replicates. Error bars represent ± SEM. Statistical significance was calculated with Student's *t*-test (*P < 0.05). **E)** Electrolyte leakage in WT and *asbbx28-1* of cold-acclimated (5 d of 4 °C) plants. Each data point represents the mean from at least 3 biological replicates (±SEM). The dashed line represents the threshold value, 50%. The dotted lines represent the curve fit. Statistical significance was calculated with an extra sum-of-squares *F-*test and the *P-*value is shown in the figure.

To further elaborate their role, the promoter region of the antisense transcription was targeted with the CRISPR-Cas9 approach to minimally alter and knockout only parts of their regulatory sequence and/or the 5′-end of the TSS window. That way, the direct interference with sense transcription would be minimized. For *ZAT5*, we were able to retrieve a mutant with a 283 bp deletion, i.e. 392 bp from the start of the TSS window of *asZAT5* and 710 bp from the poly(A) site for *ZAT5* (Fig. 9A). We named this mutant *aszat5-1*. For *BBX28*, we aimed to delete the 5′-end of the TSS window of *asBBX28*. We retrieved a 392 bp deletion that included a deletion of 220 bp of the TSS window (*asbbx28-1*, Fig. 9A). The deletion is 193 bp from the poly(A) site of sense *BBX28*. In both mutants, we did not alter the stability of the respective mRNA, suggesting that we did not interfere with transcription termination (Supplementary Fig. S5, A and B).

In *aszat5-1*, there was a significant downregulation of the nascent transcription of *asZAT5* at 22 °C but we could not detect any difference after 3 h 4 °C (Fig. 9B). For *ZAT5*, the mutant showed lower nascent transcription at both time points (Fig. 9B). The steady-state levels of *ZAT5* showed a decreased level after cold treatment (Fig. 9B). These results corroborate the results from the *ZAT5* constructs and suggest 2 key regulatory aspects, antisense transcription over the *ZAT5* locus is required for proper cold induction and that CRISPR-Cas9 targeted *asZAT5* promoter sequences contain regulatory elements in the antisense promoter to regulate the initiation of *asZAT5* transcription. To show a biological role for *asZAT5*, we performed a cold acclimation and freezing test in the *aszat5-1* mutant (Fig. 9C). We found a significantly lower freezing tolerance for the *aszat5-1* (−4.8 ± 0.2 °C, *P* = 0.0021) compared with WT (−5.9 ± 0.2 °C). In *asbbx28-1*, we detected a significant downregulation of the nascent transcription of *asBBX28* at 22 °C and lower active transcription of *BBX28* at both 22 °C and 3 h 4 °C (Fig. 9D). The steady-state level of the sense *BBX28* transcript was also lower in this mutant (Fig. 9D), corroborating that antisense transcription had a positive role on sense *BBX28* transcription as it had on *ZAT5* transcription. Additionally, we could see a significant decrease in the freezing tolerance in the mutant (−4.0 ± 1.1 °C, *P* = 0.0015 for *asbbx28-1* and −5.9 ± 0.2 °C for WT, Fig. 9E).

Our results suggest that the antisense transcription of *ZAT5* and *BBX28* has a positive role in priming the sense transcription for stress response and that the characterized antisense transcription has an important biological role in the cold acclimation process. All in all, our study shows that antisense transcription can play a crucial role in priming certain plant stress-responsive TFs.

## Discussion

A remarkable finding in our study is that the nascent transcriptional response to cold differs from the one detected by steady-state level measurements (Fig. 3). Additionally, we show that plaNET-seq complements steady-state methods (RNA-seq) to detect genome-wide transcriptional changes in the cold response (Fig. 3). A similar concept has earlier been proposed for heat stress in Arabidopsis (Liu et al. 2021). Thus, these reports highlight the importance of taking into account both active transcription and steady-state levels of mRNA to fully understand the response to stress (Sidaway-Lee et al. 2014). Changes in the active transcription of a gene measured by plaNET-seq have biological relevance, even though the steady-state level of the gene's mRNA is only slightly different to control conditions after cold exposure (Figs. 4 to 5). This report takes active transcription into account to find cold-responsive genes in Arabidopsis.

This study characterizes genes with antisense transcription that initiates from the 3′-end of genes in Arabidopsis (Figs. 1 to 2). Notably, we find a stark contrast in how the Arabidopsis genome implicates antisense transcription compared with other eukaryotes. In human cells, antisense transcription is most prevalent from early exon-intron junctions of genes (Brown et al. 2018). This type of antisense transcription is generally associated with low active transcription (Mayer et al. 2015), albeit with higher stability of the sense transcript (Brown et al. 2018). In Arabidopsis, we see almost the opposite scenario. Antisense transcription is most prevalent from the 3′-end of genes and their host genes are associated with high active transcription and fast turnover rates (Fig. 2) (Kindgren et al. 2019). In agreement, accelerated transcript degradation is suggested as an advantageous evolutionary strategy to facilitate genome-wide swift responses during cold stress in Arabidopsis (Chiba et al. 2012). It is likely that plants, being sessile organisms, have evolved distinct ways of gene regulation compared with other eukaryotes, especially when responding to biotic and abiotic stresses. This hypothesis is corroborated by the fact that many genes with antisense transcription in Arabidopsis are stress-regulated TFs (Fig. 1), proteins that are required to kick-start stress responses.

Our data paint a picture of how plants keep their stress-responsive TFs in a constant “on” mode to prime their response to stress situations. A tempting hypothesis would be that antisense transcription is involved in sense transcript degradation, a generalized regulatory role that was widely postulated for several thousands of NATs in Arabidopsis and other plant species (Borsani et al. 2005; Held et al. 2008). However, our data do not endorse the concept of “universal gene silencing roles of NATs” but rather indicates that antisense transcription could have a more positive regulatory role on sense transcription (Reis and Poirier 2021). When antisense transcription is downregulated after cold exposure (Fig. 6 and Supplementary Fig. S2), sense transcript stability is lower compared with 22 °C (Fig. 5) and in our CRISPR-Cas9 deletion lines that exhibit reduced antisense transcription, we detected decreased sense mRNA levels at 4 °C (Fig. 9). In addition, our data from *ZAT5* and *BBX28* highlight that even a marginal reduction of their antisense transcription can impair the cold-responsiveness and ability of plants to acclimate to cold temperatures (Fig. 9).

Thus, an outstanding question and an important avenue for future research is how antisense transcription could relay a positive role to mRNA steady-state levels. A possible mechanism could be that the antisense transcription increases the stability of the sense transcript and assists in the translation of the sense transcript, as shown for the rice lncRNA, *cis-NAT PHOSPHATE1;2* (*cis-NAT PHO1;2*) (Jabnoune et al. 2013). However, this is unlikely to be a general mechanism since most antisense transcripts are short-lived and degraded soon after their synthesis (Fig. 8) (Kindgren et al. 2019; Thieffry et al. 2020) and in our CRISPR lines, we did not see any effect of the mRNA stability (Supplementary Fig. S5). Another, more likely mechanism, could be antisense-promoted changes to the local chromatin environment which could be important in priming the optimal transcriptional response, resembling the example of *NUCLEAR ENRICHED ABUNDANT TRANSCRIPT 1* (*NEAT1*) lncRNA in animals where the act of transcription itself at the *NEAT1* locus was shown to be sufficient for biological function (Mao et al. 2011). It is possible that sense–antisense promoters can act autonomously both during developmental transitions and in stress conditions if uncoupled from the genomic context of each other, as shown for e.g. *asDOG1*, *COOLAIR*, and *SVALKA* (Swiezewski et al. 2009; Fedak et al. 2016; Kindgren et al. 2018). In contrast, the strong influence of native antisense transcription in *cis* over *ZAT5* and *BBX28* suggests that genomic proximity of the sense–antisense pairs could be crucial for the cold acclimation process (Fig. 9). In fact, genome-wide native transcriptional analysis in *Saccharomyces cerevisiae* reinforces the idea that the dynamic chromatin structures could be central in determining the landscape of eukaryotic sense–antisense transcription (Murray et al. 2015).

In Arabidopsis, the H3K27 demethylase RELATIVE OF EARLY FLOWERING-6 (REF6) has been proposed to recruit a chromatin remodeling complex that includes BRAHMA to regulate antisense transcription (Li et al. 2016; Archacki et al. 2017). Other repressing chromatin mechanisms have been described for *COOLAIR*, *AGAMOUS INTRONIC RNA-4* (*AG-incRNA4*), and *AUXIN PROMOTER REGULATED LOOP* (*APOLO*), albeit their mechanisms of action are distinct (Ariel et al. 2014, 2020; Csorba et al. 2014; Wu et al. 2018). In a parallel manner, it is possible for lncRNAs to mediate the deposition of activating marks in histones by recruiting other sets of modifiers, as has been shown for the antisense transcript of *MADS AFFECTING FLOWERING 4* (*MAF4*) and *MARNERAL SILENCING* (*MARS*) in Arabidopsis (Zhao et al. 2018; Roulé et al. 2022). Similar modes of action have been reported in other plant species as well, for example, an antisense lncRNA, transcribed from *DgTCP1* [*CLASS I TEOSINTE BRANCHED1/CYCLOIDEA/PROLIFERATING* (*TCP*) TF], arbitrates histone modification depositions at the sense promoters playing a positive role in cold tolerance in *Chrysanthemum morifolium* (Li et al. 2022). It will be central to focus on different histone marks and histone variants in future studies of antisense genes to elucidate noncoding transcription and its broader role(s) in plants.

Our discoveries take the first step to a broader role of antisense transcription in plants and support the notion that transcription from the complementary strand modulates the responsiveness of stress genes. Furthermore, our study moves away from the paradigm that antisense transcription has a prominent silencing role in plants instead supporting a more positive regulatory function.

## Materials and methods

### Plant growth, mutants and CRISPR-Cas9 mutant generation

For the WT background Arabidopsis (*A. thaliana*) *Col-0* or Columbia accession was employed. For the growth of plants, seeds were surface sterilized and stratified for 2 to 4 d at 4° C in the dark and either transferred to soil directly or plated on ½ Murashige and Skoog (MS) basal medium supplemented with 1% (w/v) sucrose. Plants were grown in long-day conditions [16 h light, 8 dark, ∼100 µE, SciWhite LEDs (Percival)] for 10 d. Biological replicates in all experiments represent approximately 20 to 30 seedlings grown on separate plates. Cold treatment (4° C, ∼25 µE) was initiated at ZT4 to replicate the conditions set by the plaNET-seq dataset. T-DNA insertional mutant lines, viz *zat5–1* (*SALK_041934*), *bbx28-1* (*SAIL_412_A09*), and *hen2-2* (*GABI_774H07*) (Lange et al. 2014) were genotyped and confirmed for homozygosity by PCR. For the CRISPR-Cas9 mutants, guide RNAs (gRNAs) were designed using the CHOPCHOP webserver (http://chopchop.cbu.uib.no/) and a 2gRNA fragment was amplified using DT1T2 plasmid (Xing et al. 2014) template using Phusion DNA polymerase (Thermo Fisher Scientific). Oligos used can be found in Supplementary Data Set 6. The PCR product was electrophoresed, and gel purified followed by GreenGate reaction into a modified pHSE401 binary vector as described before (Xing et al. 2014). In the modified pHSE401 vector, the hygromycin resistance has been replaced by a *GFP* seed coat expression cassette for faster screening. Final plasmids were verified by sequencing and transformed into WT *Col-0* plants by *A. tumefaciens* (*GV3101*) floral dip. T1 seeds were first selected by visual screening for *GFP* expression followed by PCR genotyping. A further selection of the T2 lines was performed by picking seeds lacking the GFP signal, and then homozygous plants were confirmed by PCR for the genomic deletion and the absence of the Cas9 construct. Seeds from homozygous plants were used in experiments.

### Generation of reporter constructs

We used GreenGate cloning system for generation of firefly luciferase (LUC) gene reporter constructs (Lampropoulos et al. 2013). To construct *ProZAT5:ZAT5-LUC-UTR-ASProZAT5* and *ProZAT5:ZAT5-LUC-tNOS*, 2,438 bp fragment upstream (1,408 bp promoter and 1,030 CDS of *ZAT5* without stop codon) was PCR amplified from genomic DNA. Separate PCR amplification was carried out for 1,402 bp long *UTR-_AS_ZAT5_prom_* fragment using proofreading Phusion DNA polymerase using a genomic DNA template. LUC and tNOS terminator (tNOS) were also separately PCR amplified in a similar manner. Subsequently, different PCR products were cloned into respective GreenGate entry modules by employing BsaI/T4 DNA ligase and using reaction conditions as described earlier (Lampropoulos et al. 2013). All GreenGate entry plasmids were confirmed by restriction digestion and DNA sequencing. Finally, the GreenGate reaction was performed for the assembly of 6 entry modules to create final destination plasmids, i.e. *ProZAT5:ZAT5-LUC-UTR-ASProZAT5* and Pro*ZAT5:ZAT5-LUC-tNOS* using pGGZ003 as backbone vector according to the protocol reported earlier (Lampropoulos et al. 2013). Oligos used can be found in Supplementary Data Set 6.

### Transient agroinfiltration and luciferase assay

*Agrobacterium* strain *GV3101* was separately transformed with plasmids harboring *_prom_ZAT5::ZAT5-LUC-UTR-ASZAT5_prom_* and *_prom_ZAT5::ZAT5-LUC-tNOS* constructs and plated on LB media containing 10 µg/ml rifampicin, 25 µg/ml gentamicin, and 75 µg/ml spectinomycin. After 48 h of growth positive colonies were selected and grown in 5 ml of LB medium supplemented with antibiotics. Additionally, the presence of constructs in *Agrobacteria* was confirmed by colony PCR. After overnight growth at 30 °C, 30 ml of fresh induction media (LB supplemented with 10 mm MES pH 5.6, 20 μM acetosyringone, 25 µg/ml, gentamicin, 75 µg/ml spectinomycin, and 10 µg / ml rifampicin) inoculated. When OD_600_ reached 0.5, cells were harvested by centrifugation and resuspended in infiltration media (LB supplemented with 10 mm MES pH 5.6, 10 mm MgCl_2_, and 150 μM acetosyringone without antibiotics) to obtain an OD_600_ value of 1 followed by incubation of bacterial culture at room temperature for a minimum of 3 h. Finally, leaves from 6-wk-old *N. benthamiana* plants were infiltrated with bacterial suspension and the area was delimited and marked with a marker.

Seventy-two hours post agroinfiltration, half of the randomly selected *N. benthamiana* plants (a minimum of 5 independent plants each with 2 to 3 infiltrated leaves for individual constructs) were subjected to cold stress treatment (3 h cold 4 °C) at ZT4. Control plants were maintained at 22 °C. Immediately after 3 h of cold stress, previously infiltrated leaves with marked areas from cold-treated and control plants were carefully and quickly re-infiltrated with 5 mm working solution of D-Luciferin (GoldBio). The ratio of 5 mM d-Luciferin in 0.01% (v/v) Triton X-100 and sterile water was kept at 1:3 during re-infiltration of leaves. Further, several 1 cm leaf discs were prepared from marked re-infiltrated area and subjected for luminescence measurement after 5 to 10 min of incubation at respective temperatures by GloMax Navigator Microplate Luminometer. Data were extracted and analyzed using Excel and GraphPad software.

### Electrolyte leakage assay

Electrolyte leakage measurements were carried out according to a previous report (Kindgren et al. 2015). In short, plants were grown in short days (8 h light /16 h dark cycle) for 4 w. For the cold acclimation experiments, WT and mutant plants were transferred to a cold chamber set at 4 °C for 4 d without changing photoperiodic conditions. Randomized leaf discs of 1 cm diameter, for each genotype in triplicates from several similar-sized leaves, were prepared using a cork borer for acclimated or non-acclimated plants and carefully placed horizontally in a manner to avoid floating in clean glass tubes filled with 200 µl deionized distilled water. The tubes containing 2 leaf discs were then transferred to a programmable freezing bath (FP51, Julabo, Germany) set at −2° C. After 45 min, icing was induced manually in each tube with the help of liquid N_2_ and a metallic stick. Temperature decrease occurred at the rate of −1 °C per 30 min, and samples were taken out at designated temperature point(s) followed by incubation on ice for at least 1 h in the cold room (4 °C). Soon after the collection and 1 h ice-incubation of tubes, 1.3 ml of water was added to each tube and placed on a shaker overnight at 4° C and conductivity was measured using a conductivity cell (CDM210, Radiometer, Denmark) on the next day. Finally, all tubes were subjected to flash freeze using liquid N_2_ and left on a shaker overnight at room temperature. To obtain the total electrolyte content from leaf discs, conductivity was measured again, and the % of electrolyte leakage was calculated using the formula – (conductivity before flash freeze/conductivity after flash freeze) * 100. Data were fitted into a sigmoidal dose–response curve using GraphPad Prism software and significant differences in the fit were determined with an extra sum-of-squares *F* test.

### RNA extraction, cDNA synthesis, and RT-qPCR

Total RNA extraction from plant material was carried out using RNeasy Plant Mini Kit (QIAGEN) as per suppliers’ instructions. The extracted RNA was additionally treated with dsDNase (Thermo Fisher Scientific) for the elimination of genomic DNA contamination. Successively, cDNA synthesis was carried out using Superscript IV (Invitrogen) reverse transcriptase as per manufacturer’s instructions using strand-specific RT primers carrying a sequence tag (GACTGGAGCACGAGGACACT) at 5′ end (Parent et al. 2015; Kindgren et al. 2018) together with a reference gene. Quantitative real-time PCR (RT-qPCR) was performed on CFX96 and CFX384 Real-Time PCR detection systems (Bio-Rad) using SYBR premix (Bio-Rad), cDNA, reverse primer (aligning to tag sequence), and appropriate forward primers at the concentration of 10 pmol/µl with the PCR cycler following initial denaturation at 95 ° C for 30 s, standard 40 cycles of 94 ° C for 10 s and 60 ° C for 30 s. The specificity of RT-qPCR products was assessed from the single peak melt curves. For the data analysis, the *C_q_* values from a minimum of 3 biological replicates with 2 to 3 technical replicates were averaged and △*C_q_* was obtained as *C_q_* (gene of interest) − *C_q_* (reference gene). Final calculations were performed by 2^(−Δ*c_q_*)^ or 2^(−ΔΔ*c_q_*)^, adjusted to experimentally determined primer efficiency for determination of fold change in gene expression levels. Statistical significant differences were calculated with Student's *t*-test. Primers used are listed in Supplementary Data Set 6.

### Measuring nascent transcription

Nuclei were isolated from around 3 g of 12-d-old seedlings with Honda buffer (0.44 m sucrose, 1.25% Ficoll, 2.5% dextran T40, 20 mm Tris–HCl pH 7.4, 10 mm MgCl_2_, 0.5% Triton X-100, Prot. inhibitor tablet, RNase inhibitor, and 5 mm DTT). The nuclear lysis and RNAPII-IP were done according to Kindgren et al. (2018) with small modifications. Briefly, after lysis and DNAse I treatment, the supernatant was mixed with protein G magnetic beads (Thermo Fisher Scientific) coupled to an endogenous RNAPII antibody (8WG16, Sigma Aldrich) for 2 h at 4 °C. The beads were washed 4 times with wash buffer [0.3 M NaCl, 20 mM Tris–HCl pH 7.5, 5 mM MgCl2, 5 mM DTT, proteinase inhibitor tablet, and RNase inhibitor (20 U/ml)]. To disrupt the RNAPII complexes, QIAzol was added, and RNA was isolated using the miRNeasy kit from Qiagen. RNA concentration was measured with Nanodrop and approximately 100 ng was used for cDNA synthesis with gene-specific primers and Superscript IV (Invitrogen) according to manufacturer's instructions. Oligos used can be found in Supplementary Data Set 6.

### RNA-seq and analysis

RNA was isolated from 10-d-old Arabidopsis Col-0 seedlings grown on ½ MS medium. Briefly, seeds were stratified for 2 to 4 d at 4 °C in the dark, followed by growth in a long day (16 h light/8 h dark, 22 °C day/18 °C night) conditions and ∼100µEm^−2^s^−1^ light. On the 12th day, seedlings were subjected to cold stress (4 °C) for 3 and 12 h with ∼20 to 25µEm^−2^s^−1^ light. Total RNA was isolated using RNeasy Plant Mini Kit (QIAGEN) according to manufacturer's instructions. RNA thus obtained was treated with TURBO DNAse (Thermo Fischer Scientific) according to the standard protocol. Three biological replicates from each time point of RNA samples were sent to Novogene Europe where strand-specific libraries were prepared and sequenced using Illumina's NovaSeq 6000 platform. Libraries were sequenced to a depth of 40 to 60 million raw reads (6G raw data per sample). For data analysis, the guidelines previously established at UPSC were followed (Delhomme et al. 2023). Pre-processing of data was done using FastQC v0.11.9 (quality control of the raw data) and SortMeRNA v4.3.4 (Kopylova et al. 2012; filter and remove rRNA contamination). Thereafter, Trimmomatic v0.32 (Bolger et al. 2014) was used to trim the adapter sequences and FastQC was performed again to ensure data integrity. Salmon v1.6.0 (Patro et al. 2017) was used to determine the read counts with ARAPORT11 as a reference. R-package DESeq2 (Love et al. 2014) was used to perform the differential expression analysis. Statistically significant genes were filtered using the following parameter: false discovery rate < 0.05 and log_2_ fold change ≥ 0.5. RNA-seq data have been deposited on GEO (GSE252832). To investigate the rate of transcript degradation of control genes vs. PAS-host genes, we used the Decay Rate (alpha estimate) from Sorenson et al. (2018) (Table S2 from cited paper), which was originally determined by cordycepin treatment followed by RNA-seq. In addition, we used available data to estimate the decay rate for mRNAs after actinomycin D treatment (Narsai et al. 2007). Gene Ontology (GO)-term enrichment was done at TAIR (https://www.arabidopsis.org/tools/go_term_enrichment.jsp).

### RNA stability assay

RNA stability measurements to determine the half-life (*t*_1/2_) of transcripts were performed according to previous report (Fedak et al. 2016). In summary, 10-d-old WT seedlings were grown in long day photoperiod (16 h light/8 h dark, 22 °C day/18 °C night) (CLF Plant Climatics cabinet) over ½ MS medium supplemented with 1% sucrose (w/v). On Day 10, seedlings were transferred to liquid ½ MS media and acclimatized while maintaining 22 °C or 4 °C growth temperatures for respective sample sets under the same light conditions. Further, samples from 22 °C or 4 °C were transferred to incubation buffer (1 mm PIPES at pH 6.25, 1 mm trisodium citrate, 1 mm KCl, and 15 mm sucrose) in 12-well plates for 30 min followed by the addition of 150 mg/l cordycepin (3′-deoxyadenosine; Sigma Aldrich). Immediately after cordycepin addition, samples were vacuum infiltrated for (5 min × 2 times). 15 seedlings for each of the samples in triplicates were collected at 0, 15, 30, 60, and 120 min after cordycepin treatment. Subsequently, total RNA extraction and strand-specific RT-qPCR analyses were carried out by using cDNA as a template synthesized with SuperScript IV Reverse Transcriptase (Invitrogen) and gene-specific primers. *EIF4A1* (*AT3G13920*) (Perea-Resa et al. 2012) was used as an internal assay control. *C_q_* values at 15, 30, 60, and 120 min were normalized with *C_q_* at 0 min, and RNA degradation curve was obtained by [*C_q_*(*n*) = ln (*C_q_*/*C_q_* (0)) * (−10)] equation. Finally, *t*_1/2_ of transcripts was calculated from the obtained slope [*t*_1/2_ = (ln_2_)/slope]. Oligos used can be found in Supplementary Data Set 6.

### Genome-wide analyses

Detailed bioinformatics methods can be accessed at https://github.com/peterkindgrengroup/Meena_et_al_2024. Briefly, a control set of genes without PAS was curated from all expressed genes (22 °C RNA-seq data from this study). In order to better define the gene coordinates of both controls and PAS-host genes, we used available TSS-seq data (S4 Table from cited paper) (Nielsen et al. 2019) in combination with the TTS from TAIR10. Gene length was calculated from these curated genomic features. RNAPII datasets were retrieved (GSE95301 at Gene Expression Omnibus) (Liu et al. 2018). Bedgraphs from samples GSM2522253 for PolII_WT were converted to bigwig (Kent et al. 2010). DeepTools (Ramírez et al. 2016) was used to compute ChIP-seq score matrices and to generate metaplots along the gene body. Differentially expressed genes from plaNET-seq were extracted from Supplementary Table S2 (Kindgren et al. 2019). To build the plaNET-seq metaplots, the raw sequences (SRR9117170-SRR9117181) were downloaded and processed as indicated in the previously mentioned GitHub repository. Shortly, after the quality control of raw reads, bam files were generated by aligning the sequence reads against the Arabidopsis genome using STAR 2.7.10a (Dobin et al. 2012) and used on ngs.plot (Shen et al. 2014) to generate the meta-gene profiles using the in-built TAIR10 genome and the gene lists of interest. A few existing datasets were remapped for this study. They include DR-seq (Schurch et al. 2014), CAGE (Thieffry et al. 2020), and plaNET-seq (Kindgren et al. 2019).

### Accession numbers

Arabidopsis Genome Initiative locus identifiers for the genes mentioned in this article are as follows: At1g18710 for *MYB47*, At1g73870 for *COL7*, At2g28200 for *ZAT5*, At5g52510 for *SCL8*, and At4g27310 for *BBX28*.

## Supplementary Material

koae160_Supplementary_Data

## Data Availability

RNA-seq data for WT is available online at NCBI under accession number GSE252832. All new code is available at https://github.com/peterkindgrengroup/Meena_et_al_2024. Statistical data are provided in Supplementary Data Set 7.

## Supplementary data

The following materials are available in the online version of this article.

**Supplementary Figure S1.** Half-life and RNAPII occupancy of PAS genes.

**Supplementary Figure S2.** PAS transcription in response to cold temperature.

**Supplementary Figure S3.** RNA level of *ZAT5* and *BBX28* with control at different ZT and freezing test of non-acclimated *zat5-1* and *bbx28-1* plants.

**Supplementary Figure S4.** LUCIFERASE assay for *ZAT5* constructs.

**Supplementary Figure S5.** RNA stability of *ZAT5* and *BBX28* in CRISPR mutants.

**Supplementary Data Set 1.** List of PAS genes.

**Supplementary Data Set 2.** Full list of GO terms.

**Supplementary Data Set 3.** Differentially expressed genes in the RNA-seq experiment.

**Supplementary Data Set 4.** Differentially expressed PAS genes.

**Supplementary Data Set 5.** PAS genes with similar expression profile to *CBF2*.

**Supplementary Data Set 6.** Oligos used in this study.

**Supplementary Data Set 7.** Statistical data.

## Dive Curated Terms

The following phenotypic, genotypic, and functional terms are of significance to the work described in this paper:

REF6 Gramene: AT3G48430
REF6 Araport: AT3G48430

clf Gramene: AT2G23380
clf Araport: AT2G23380

EIF4A1 Gramene: At3g13920
EIF4A1 Araport: At3g13920

HYH Gramene: AT3G17609
HYH Araport: AT3G17609

WRKY48 Gramene: AT5G49520
WRKY48 Araport: AT5G49520

MAF4 Gramene: AT5G65070
MAF4 Araport: AT5G65070

AT2G28200 Gramene: AT2G28200
AT2G28200 Araport: AT2G28200

AT3G13920 Gramene: AT3G13920
AT3G13920 Araport: AT3G13920

AT4G27310 Gramene: AT4G27310
AT4G27310 Araport: AT4G27310

CBF2 Gramene: AT4G25470
CBF2 Araport: AT4G25470

CBF1 Gramene: AT4G25490
CBF1 Araport: AT4G25490
