# Supplementary material for: Antisense transcription from stress-responsive transcription factors fine-tunes the cold response in Arabidopsis
Source: Plant Cell. 2024 May 27;36(9):3467–82. doi: 10.1093/plcell/koae160 (PMC11371176; doi:10.1093/plcell/koae160)
Supplement: koae160_Supplementary_Data [file koae160_supplementary_data.zip › Supplemental Data.pdf]

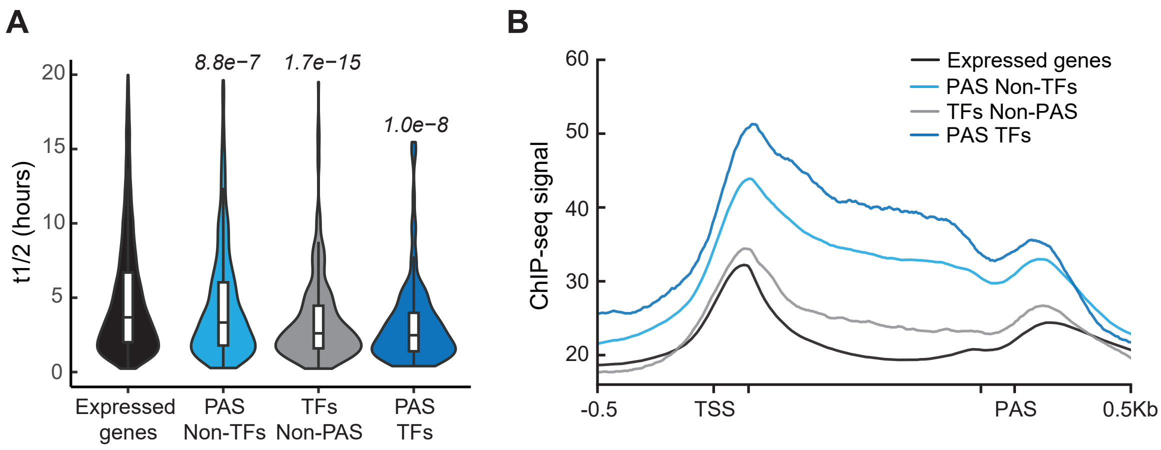

Supplementary Figure S1 (Supports Figure 1).
A) Violin plot of the decay rate of PAS non-TF genes, TFs non-PAS, PAS TF genes and all expressed genes after transcriptional inhibition by actinomycin D. p value was calculated with Mann-Whitney U test.
B) Metagene analysis of ChIP-seq data (RNAPII) of PAS non-TF genes (light blue), TFs non-PAS (grey), PAS TF genes (blue) and all expressed genes (black). The shaded area shows a 95% confidence interval for the mean.

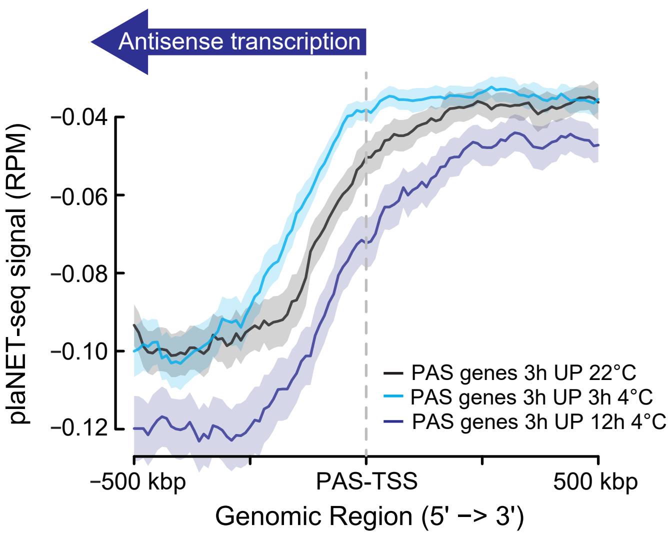

Supplementary Figure S2 (Supports Figure 4).
Metagene analysis of the plaNET-seq signal in a 1 kb window centered at the PAS-AS TSS. 22°C is shown in black, 3 h 4°C in cyan, 12 h 4°C in blue. The shaded area shows 95% confidence interval for the mean.

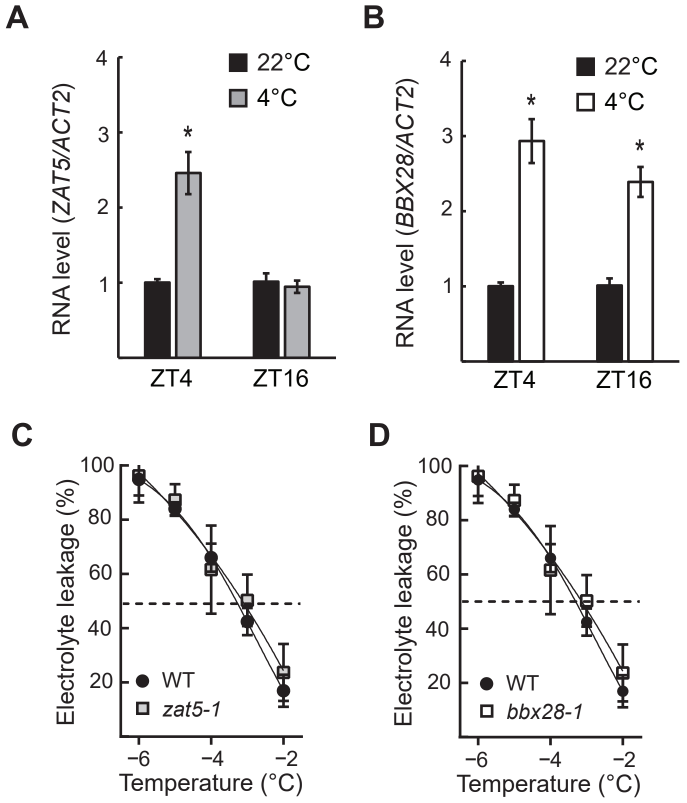

Supplementary Figure S3. RNA level of *ZAT5* and *BBX28* with control at different ZT and freezing test of non-acclimated *zat5-1* and *bbx28-1* plants. (Supports Figure 7).

A) The relative steady-state level of *ZAT5* in wild-type measured by RT-qPCR at 22°C and following 3h and 12h of cold exposure (4°C). The 22°C samples were taken at the same time point as the cold samples. All levels were normalized to WT levels at 22°C. The mean values are from three biological replicates. Error bars represent ± SEM. Statistical significance was calculated with Student's t-test (* $p<0.05$).

B) The relative steady-state level of *BBX28* in wild-type measured by RT-qPCR at 22°C and following 3h and 12h of cold exposure (4°C). The 22°C samples were taken at the same time point as the cold samples. All levels were normalized to WT levels at 22°C. The mean values are from three biological replicates. Error bars represent ± SEM. Statistical significance was calculated with Student's t-test (* $p<0.05$).

C) Electrolyte leakage in wild-type and *zat5-1* of non-acclimated plants. Each data point represents the mean from at least 3 biological replicates (±SEM).

D) Electrolyte leakage in wild-type and *bbx28-1* of non-acclimated plants. Each data point represents the mean from at least 3 biological replicates (±SEM).

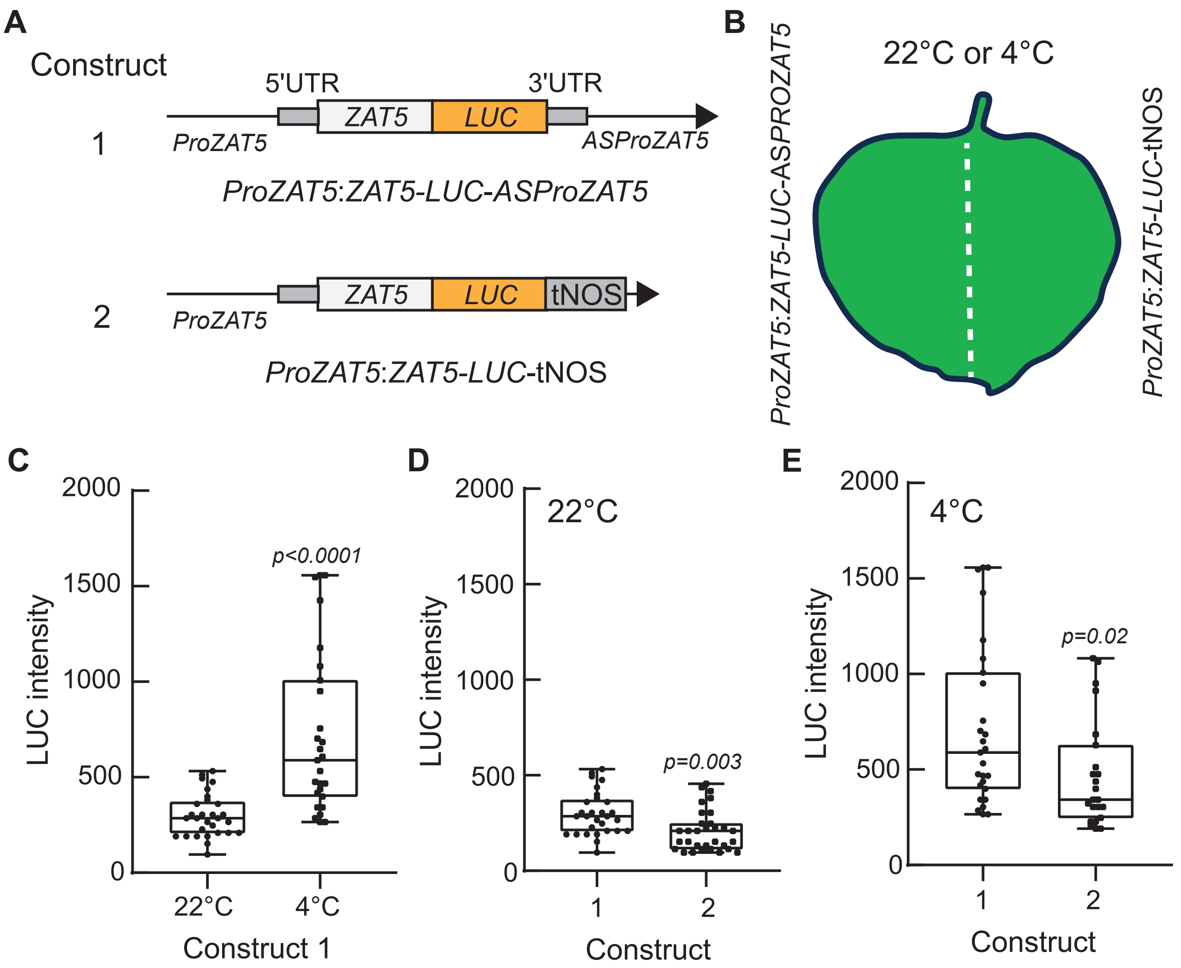

Supplementary Figure S4. LUCIFERASE assay for *ZAT5* constructs. (Supports Figure 8).
A) Luciferase reporter assay for *ZAT5*. The gene schemes are shown for construct 1 and 2. Full length construct 1 contains *ZAT5* promoter, CDS and 1402 bp long 3' UTR and *ASZAT5* promoter. In construct 2, the 3' UTR-antisense-promoter part is replaced by tNOS terminator to prevent antisense transcription.
B) Scheme of transient infiltration in N. benthamiana leaf. Each individually infiltrated leaf contained construct 1 and 2 on left and right side, respectively for both 22° C and 4°C conditions.
C-E) Box plots showing LUC intensity for **C)** construct 1 at 22° C and after 3 hours of cold stress at 4°C. Comparative LUC intensity for construct 1 and 2 at **D)** 22° C and **E)** at 4°C. Box plots show data points, centre value: median, box limits: upper and lower quartiles, whiskers: 5th to the 95th percentile. Statistically significant differences compared to wild type were evaluated by t-test. p values are shown on top of plots.

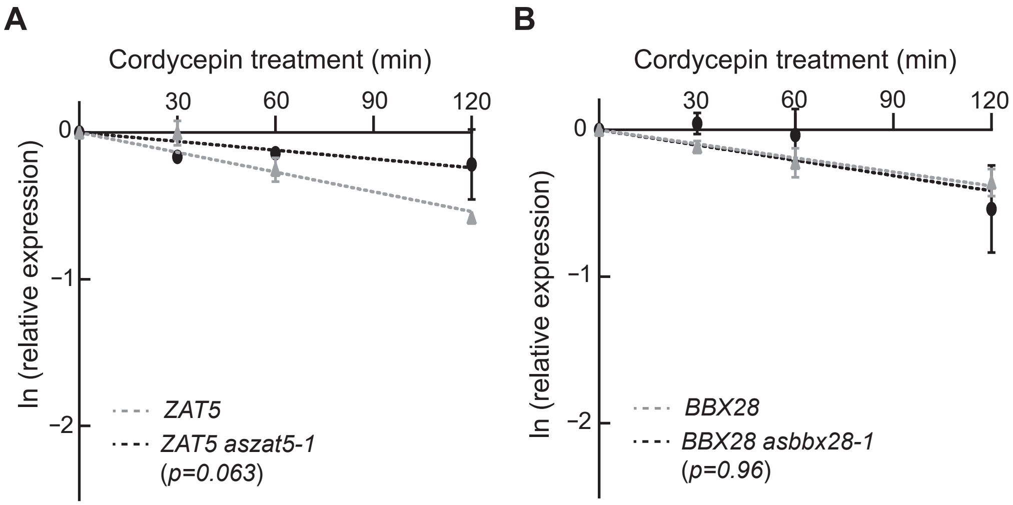

Supplementary Figure S5. RNA stability of *ZAT5* and *BBX28* in CRISPR mutants. (Supports Figure 9). A-B) Transcript stability assays for **A)** *ZAT5*, and **B)** *BBX28* in respective CRISPR-Cas9 mutant lines (described in Figure 9) and in wild type after transcriptional inhibition with cordycepin at 22°C and 4°C. Half-life (t1/2) was determined from the slope of degradation curves that were obtained after RT-qPCR analysis of cordycepin treated seedlings at indicated time points. Each data point is the mean of three biological replicates. Error bars represent ± SD.
