# Supplementary material for: Antisense transcription from stress-responsive transcription factors fine-tunes the cold response in Arabidopsis
Source: Plant Cell. 2024 May 27;36(9):3467–82. doi: 10.1093/plcell/koae160 (PMC11371176; doi:10.1093/plcell/koae160)
Supplement: koae160_Supplementary_Data [file tpc.00032.2024-s11.pdf › tpc.00032.2024-s11.pdf]

THE PLANT CELL

Antisense transcription from stress responsive transcription factors fine tunes the cold response in Arabidopsis thaliana: insights from BBX28 and ZAT5 characterization.

Shiv Kumar Meena, Marti Quevedo, Sarah Muniz Nardeli, Clément Verez, Susheel Bhat, Vasiliki Zacharaki, and Peter Kindgren

Review Timeline:

Submission Date:	01-Sep-2022
Editorial Decision:	22-Oct-2022
Resubmission Received:	12-Jan-2024
Editorial Decision:	18-Feb-2024
Revision Received:	22-Mar-2024
Editorial Decision:	13-Apr-2024
Revision Received:	18-Apr-2024
Accepted:	27-Apr-2024

Dr. Peter Kindgren
Umeå Plant Science Centre
Umeå
Sweden

Dear Dr. Kindgren:

Thank you for choosing to send your manuscript entitled "plaNET-seq reveals genes hosting antisense transcripts involved in the early cold stress response in Arabidopsis thaliana." for consideration at The Plant Cell. Your submission has been evaluated by members of the editorial board as well as expert reviewers in your field, and we regret to inform you that we are not able to recommend the publication of this manuscript in its current form. We have had input from multiple scientists and we have solicited post-review comments.

That said, we would be willing to re-consider a new manuscript that fully addressed the concerns raised during this review process. If you decide to resubmit to The Plant Cell, it will be evaluated as a new submission subject to full assessment by the editorial board, and if sent for external review, a new set of reviewers may be chosen if current reviewers are unavailable or the editors feel it is appropriate. Reviewers will be asked to assess your work as a new manuscript (i.e. are the claims fully supported by the data and do the results presented move the field forward?), and not only whether previous reviewer comments have been addressed.

While there was a general consensus that the study is interesting and carries potential for novelty, particularly in the detailed follow-up on the roles of ZAT5 and BBX28, the reasoning behind why these two genes were selected needs to be clarified and datasets need to be provided as well as statistically summarized in order to assess the validity of the primary claims. Provision of the datasets that form the foundation for analysis in a clear, well-documented manner is also particularly important.

It will be important to convince the editors and reviewers that the study adds significant new understanding(s) of mechanisms or processes, or otherwise represents a significant advance in the field, and that the major claims made are fully justified by the data presented. This includes careful consideration and explanation of the various controls used in experiments, the extent and manner of replication, and the statistical analyses used. Sampling methods and the nature of "biological replicates" should be described precisely (i.e. different plants, parts of plants, pooled tissue, independent pools of tissue, sampled at different times, etc.), along with a clear description of and rationale for any statistical analyses conducted. The reader should know exactly what was sampled; what forms the basis of the calculation of any means and other statistical variables and parameters reported. This is also necessary to ensure that proper statistical analysis was conducted.

The Plant Cell now requires authors to complete and submit an author revisions checklist upon submission of a revised manuscript. The aim of the checklist is to aid authors in preparing a high-quality manuscript, facilitate the review and assessment of revised manuscripts, and help to ensure that journal standards are maintained across the board. If your manuscript is accepted, the completed checklist will be published as supplemental material attached to the article online. Please download a copy of the checklist (a fillable PDF form) at this link, for submission with your revised manuscript: https://tpc.msubmit.net/html/Author_Revisions_Checklist.pdf.

Note also that supplemental materials should be restricted to large datasets and tables, presentation of replicates, and validation of reagents, methods, or genotypes. Any data that are used to support the main claims must be in the main manuscript. Supplemental figure legends must indicate what figure in the main manuscript is supported by the supplemental data presented.

We thank you for your interest in and support of The Plant Cell. We hope we will be able to render a more positive decision on future work.

Sincerely,

The Plant Cell Editorial Board

--- Reviewer comments:

Reviewer #1 (Comments for the Author):

In this manuscript, Meena et al. characterized genes with antisense transcription initiating at the Poly(A) signal (PAS genes) and

studied their roles in cold response in Arabidopsis. The authors showed that PAS genes displayed high RNA Polymerase II (RNAPII) occupancy at the genome-wide level, yet their mRNAs had a significant rapid turnover, which was unexpected. Therefore, they compared the previous plant Native Elongation Transcript sequencing (plaNET-seq) data with newly performed RNA-seq data and found that only a small fraction of differentially expressed (DE) genes overlapped between these two datasets. Interestingly, a large portion of PAS genes responded to cold according to the plaNET-seq analyses. From these cold-responsive PAS genes, the authors picked two transcription factors, namely ZAT5 and BBX28, which also showed no significant increase in the steady-state transcript levels after cold treatment, and studied their roles in cold stress response. They found out that both mutants were impaired in their acclimation to cold. They further showed that the stability of both ZAT5 and BBX28 mRNA decreased at cold temperatures. Next, the authors studied the antisense transcript from these two genes. They found out that the down-regulation of antisense ZAT5 and BBX28 mRNA led to decreased expression of ZAT5 and BBX28 and reduced freezing tolerance. Therefore, the authors concluded that plaNET-seq can more precisely illustrate biologically important genome-wide transcriptional behaviors than RNA-seq and that antisense transcription may play positive roles in abiotic stress response.

Overall, I agree with most of the authors' conclusions. More data is needed to support some of the statements. I also have some questions on data presentation and rationale. Please see my comments below.

Major points
1. It is well-known that cold stress response, especially the cold-induced gene expression in plants, is controlled by the circadian clock. I don't see any description of the ZT times when plants were treated with cold temperatures in this manuscript and the authors' previous publication in 2019 (https://doi.org/10.1093/nar/gkz1189). Therefore, a potential problem leading to the discrepancy between the RNA-seq and plaNET-seq could be that the cold treatments were not performed at the same ZT. Please clarify.
2. Figure 2C: it is hard for me to believe that the mRNA decay rate of PAS host genes was dramatically higher than non-PAS genes, although I understand that the difference was statistically significant. Please provide another way of data presentation to show that the overall mRNA decay rate is higher for PAS host genes.
3. I appreciated that the authors showed the mRNA decay rates of ZAT5 and BBX28 were higher at cold temperatures and both sense and antisense transcripts were important for freezing tolerance. However, I'm wondering how representative these results were. First, could the authors provide the rationale for selecting these two genes? Second, could the authors show that this phenomenon was prevalent with other cold-responsive PAS transcription factors? Third, it is not convincing to me that the freezing tolerance phenotype was significant. Statistical analyses were missing in Figure 5C-D and Figure 8D, H. Besides, have the authors examined the survival rates of these mutants (both the ones disrupting the sense and antisense transcripts) after freezing treatments?

Minor points:
1. Line 30: plaNET-seq was not introduced in the Abstract.
2. Line 38: the meaning of "positive role" here is obscure.
3. Figure 1B-C: can the authors clarify whether the GO terms shown here were in the top list or handpicked? It would be better if the authors showed all the enriched GO terms in a supplemental table.
4. Figure 1D: please indicate sense and antisense in the figure.
5. Line 127: please define "short genes".
6. Figure 2: "PAS host genes" were compared with "Non-PAS genes" in figure 2; however, it was described in the text and figure legends that PAS genes were compared with all expressed genes (e.g., line 129-130). Please clarify.
7. Line 143: please also define DE here (FDR, fold change, ...)
8. Line 156: define "responded".
9. Line 230: "...283 bp deletion 331 bp..." the numbers here didn't match with what was shown in Figure 8A. Please clarify.

Reviewer #2 (Comments for the Author):

Work by Shiv Kumar Meena et al. aims to use plaNETseq to uncover antisense transcription in response to cold. They then select two examples and characterise the antisense and sense transcripts.
In my view, this manuscript requires a relatively high amount of work before it is mature.

1. I have a problem with the selected genes ZAT5 and BBX28. In the text, the authors say they selected them because the genes "responded positively to cold temperatures (3h of 4{degree sign}C) in the plaNET-seq data but not in the RNA-seq data (Supplemental Data Set S5)"

but then Fig 5A and B show that those genes' steady-state RNA level is responsive to cold at similar data points as used for RNAseq?

2. If the authors are right and the BBX28 and ZAT5 nascent RNA is induced by cold but steady-state RNA is not then how can they control the phenotype? Maybe it is at later time points that the mRNA is affected? If it is not the mRNA that has the

phenotypic effect then a trans-target for nascent RNA? This is very mysterious and needs to be clarified.

3. The CRISPR-generated lines are important but:
a. they are not well characterised. At least: nascent RNA (chromatin attached) RNA level quantification of sense and sense RNA stability are needed.
b. More importantly a CRISPR deletion of a region behind a gene can have an effect on termination of the sense gene irrespective of how strong the authors stress it is not the case. So a couple of more approaches are required, like transgenes with 3' end of the sense gene exchanged for NOS or Rubisco terminators.

4. An important technical aspect is the author's ability to measure the antisense. How sure are they that what they see in qPCR is not the sense transcript? One way to distinguish would be to measure the antisense level in sense t-DNA lines. If sense (behind T-DNA) is down but antisense is not affected they are good. If antisense is affected, then of course it may be a biological effect of sense transcription on the antisense promoter firing.

I also think the authors missed several important papers:
Immediate transcriptional responses of Arabidopsis leaves to heat shock (10.1111/jipb.12990)
a paper describing nacent RNA versus stabel RNA in response to heat. So very similar to what the authors describe.

"Direct measurement of transcription rates reveals multiple mechanisms for configuration of the Arabidopsis ambient temperature response" - From PubMed Central: PMC4053849, a paper that looks at the heat effect on RNA stability and transcription. Reaching a conceptually similar conclusion about buffering of the transcription effects by RNA stability.

"Arabidopsis SWI/SNF chromatin remodeling complex binds both promoters and terminators to regulate gene expression".
Pubmed ID: PAMID 27994035
A paper describing a collection of genes with antisense transcription. Would be nice to compare the set of genes the authors described with the one described in this manuscript.

Minor points:

#90 "Nevertheless, lncRNAs have been characterized to control "
lncRNA should be changed to antisense RNA or similar

#108 "Importantly, experimental validation demonstrated that their antisense transcripts are positively correlated to the stress responsiveness of the sense transcription, indicating a general role for antisense "

Well experimental validation of 2 examples (as suggested by the previous sentence) can not indicate a general role for antisense. I recommend tuning down and saying something like: "indicated a possibility of" or "suggested that".

Reviewer #3 (Comments for the Author):

In this manuscript, Meena and colleagues seek to characterize a class of antisense RNAs previously defined based on overlap with the sense gene's polyadenylation site, PAS-associated antisense RNAs. I certainly agree that up to this point it has been difficult to generalize what functional roles antisense RNAs may play as a class, if generalizations are possible. The motivations of this study are warranted and I am happy to see work pointing in this direction. However, substantial changes and additions to this manuscript would be necessary to support the central claims.

My largest concerns revolve around a lack of clarity and transparency about the datasets that were generated and/or used in this study. Additionally, the gene editing experiments performed to test whether antisense transcription affects sense gene products do not rule out a direct impact on sense transcription.

Major questions:
1. Key pieces of information are missing that prevent me from assessing the correctness of the work. The GitHub page on line 448 (https://github.com/martiquevedo/Kumar_etal_2022) is an empty repository. No details are given about the RNA-seq libraries generated for this study. How many replicates were made? How deeply sequenced were the libraries? Without any

technical information about the quality and content of these libraries, I cannot rule out that the lower number of DEGs in RNA-seq vs. plaNET-seq in this case is simply a statistical artifact from, for example, poor within-treatment correlation.
2. Supplemental Data Set S3 reports 1441 upregulated DEGs and 1584 down at 3 hours from RNA-seq, and 2274/2509 up/down at 12 hours. These gene sets are nearly twice as large as what is depicted in the white+dark gray bars of Fig3A. Unless I am missing something, these two pieces of data should correspond?
3. The manuscript shows results from a number of public sequencing datasets without providing a proper "chain of custody". I assume that the plaNET-seq datasets referenced in Figure 1D are the same as those on GEO under accession GSE131733. But there are two reps for each timepoint there and only one track each in Fig1D, Fig4A-B, Fig7A-B, and FigS1. Were the reads pooled? If so, how? For Fig7, where did the CAGE and DRS-seq data come from? Are the tracks labeled "CAGE" the same as the Nielsen et al. 2019 [1] TSS-seq data mentioned on line 451? I found no source for the DRS-seq data in the methods or references.
4. (Fig 2D) The tendency to find PAS-associated asRNA at highly-transcribed genes could be interpreted as a consequence of extraneous polII binding due to high local polII occupancy. Across all PAS genes, are changes to asRNA transcription observed before or after changes to the sense RNA? Is the time resolution of the previous plaNET-seq experiments sufficient to determine this?
5. The gene editing experiment designed to test the effects of asZAT5 and asBBX28 cannot rule out a direct effect on sense gene transcription. Some of the earliest arabidopsis transgenics showed that sequences downstream of a gene can be essential for its expression [2]. Moreover, the plaNET-seq data in Fig4B shows that sense transcription robustly extends beyond the BBX28 PAS by ~500bp. I do not see how a large deletion 193bp downstream "exclude any potential effect on sense transcription" (line 243). To assess a causal relationship between the sense and antisense RNAs would require inducible activation or knockdown of the asRNA, such as with a pER8 promoter construct as shown by Zhao et al. 2018 [3].
6. After emphasizing that measuring steady-state RNA is unsuitable for studying the early cold stress response, the effects of the two CRISPR mutant lines are quantified by measuring steady-state RNA at single time point (Fig8B-C,F-G). Both asZAT5 and asBBX28 are still detected in their respective mutant lines; are their nascent transcripts still cold-responsive in the same was as shown in FigS2? Measuring nascent sense and antisense transcription over a time course of cold treatment in wild-type and the two CRISPR lines would be necessary to show (1) whether cold-induced changes are affected by the deletions and (2) if changes to antisense RNA transcription precede changes to sense RNA transcription. The arrow of causality cannot be determined by the results shown.

Other points:
1. (line 155) What percent of non-PAS genes respond to cold at one or both time points? Are PAS genes responsive to a degree that other genes are not?
2. (line 178) Even if it is not statistically significant, the 2 hour timepoint measurements in Fig5A are nearly 2-fold higher than at 22C, and the directions of change agree with the plaNET-seq fairly well. Measuring nascent and mature RNA simultaneously during the same time course would give more valuable insights here.
3. (line 184) A brief description of the electrolyte leakage assay here would be important to improve readability, along with an explanation of what is meant by "freezing tolerance" (i.e., LT50).
4. (line 215) Here it is not made obvious that, while both asRNAs show differences after cold, the changes are opposite to each other (asZAT5 up and asBBX28 down, FigS2A-B). How can this be reconciled with a model where both asRNAs increase sense transcription?
5. (line 411) The URL provided gives a 404 error.
6. Was the difference between nascent RNA and mature RNA abundance during the cold response not already shown in Kindgren et al. 2019 [4] by comparing plaNET-seq to TSS-seq (Fig4C)?

1. Nielsen M, Ard R, Leng X, Ivanov M, Kindgren P, Pelechano V, et al. Transcription-driven chromatin repression of Intragenic transcription start sites. PLOS Genetics. Public Library of Science; 2019;15:e1007969.
2. Larkin JC, Oppenheimer DG, Pollock S, Marks MD. Arabidopsis GLABROUS1 Gene Requires Downstream Sequences for Function. The Plant Cell. 1993;5:1739-48.
3. Zhao X, Li J, Lian B, Gu H, Li Y, Qi Y. Global identification of Arabidopsis lncRNAs reveals the regulation of MAF4 by a natural antisense RNA. Nat Commun. Nature Publishing Group; 2018;9:5056.
4. Kindgren P, Ivanov M, Marquardt S. Native elongation transcript sequencing reveals temperature dependent dynamics of nascent RNAPII transcription in Arabidopsis. Nucleic Acids Research. 2020;48:2332-47.

Response to reviewers

Reviewer #1 (Comments for the Author):

In this manuscript, Meena et al. characterized genes with antisense transcription initiating at the Poly(A) signal (PAS genes) and studied their roles in cold response in Arabidopsis. The authors showed that PAS genes displayed high RNA Polymerase II (RNAPII) occupancy at the genome-wide level, yet their mRNAs had a significant rapid turnover, which was unexpected. Therefore, they compared the previous plant Native Elongation Transcript sequencing (plaNET-seq) data with newly performed RNA-seq data and found that only a small fraction of differentially expressed (DE) genes overlapped between these two datasets. Interestingly, a large portion of PAS genes responded to cold according to the plaNET-seq analyses. From these cold-responsive PAS genes, the authors picked two transcription factors, namely ZAT5 and BBX28, which also showed no significant increase in the steady-state transcript levels after cold treatment and studied their roles in cold stress response. They found out that both mutants were impaired in their acclimation to cold. They further showed that the stability of both ZAT5 and BBX28 mRNA decreased at cold temperatures. Next, the authors studied the antisense transcript from these two genes. They found out that the downregulation of antisense ZAT5 and BBX28 mRNA led to decreased expression of ZAT5 and BBX28 and reduced freezing tolerance. Therefore, the authors concluded that plaNET-seq can more precisely illustrate biologically important genome-wide transcriptional behaviours than RNA-seq and that antisense transcription may play positive roles in abiotic stress response.

Overall, I agree with most of the authors' conclusions. More data is needed to support some of the statements. I also have some questions on data presentation and rationale. Please see my comments below.

Major points

1. It is well-known that cold stress response, especially the cold-induced gene expression in plants, is controlled by the circadian clock. I don't see any description of the ZT times when plants were treated with cold temperatures in this manuscript and the authors' previous publication in 2019 (https://doi.org/10.1093/nar/gkz1189). Therefore, a potential problem leading to the discrepancy between the RNA-seq and plaNET-seq could be that the cold treatments were not performed at the same ZT. Please clarify.

We thank the reviewer for pointing out this obvious concern and apologize if it was not clear enough in the previously submitted manuscript. Our both data sets are obtained from plants grown in same manner and conditions, a perquisite that we strictly followed before RNA-Seq analysis, and cold treatment was initiated at ZT4 for RNA-seq and plaNET-seq. We have also clarified this in the method section.

2. Figure 2C: it is hard for me to believe that the mRNA decay rate of PAS host genes was dramatically higher than non-PAS genes, although I understand that the difference was statistically significant. Please provide another way of data presentation to show that the overall mRNA decay rate is higher for PAS host genes.

We understand the reviewer's concern and agree that this was not clear enough in our previously submitted manuscript. To address this point and to make it clearer, we have divided the PAS genes into PAS transcription factors (PAS-TFs) and PAS non-TFs in Fig. 2. The main reason for this division was the enrichment of TFs in PAS host genes and our interest in cold responsive TFs. PAS-TFs show a more extreme decay rate compared to PAS non-TFs and expressed genes (control). In addition,

we have used another study (PMID: 18024567) to assess decay rates (Fig. S1A). Both data sets show an increased decay rate/lower half-life for PAS-TFs.

3. I appreciated that the authors showed the mRNA decay rates of ZAT5 and BBX28 were higher at cold temperatures and both sense and antisense transcripts were important for freezing tolerance. However, I'm wondering how representative these results were. First, could the authors provide the rationale for selecting these two genes? Second, could the authors show that this phenomenon was prevalent with other cold-responsive PAS transcription factors? Third, it is not convincing to me that the freezing tolerance phenotype was significant. Statistical analyses were missing in Figure 5C-D and Figure 8D, H. Besides, have the authors examined the survival rates of these mutants (both the ones disrupting the sense and antisense transcripts) after freezing treatments?

Thank you for your comments. We agree that our initial manuscript did not address these points properly. In our substantially enhanced manuscript, we have included three more representative mRNAs from our list of candidate genes and measured their transcript stability in 22°C and 4°C (Fig. 3) supporting our previous conclusion that this phenomenon is widespread with other cold-responsive PAS transcription factors as well. In the text, we have clarified our reasoning why we chose these genes (Line 176 and forward). The primary reason why we went forward with **ZAT5** ***and*** **BBX28** ***was that we were able to generate CRISPR deletion lines for these genes by knocking out a minimal genomic region around antisense TSS (although, we rigorously attempted to generate CRISPR knockout lines for all 5 genes in Figure 4). In addition, we have added statistical tests to the freezing tests. Our freezing tests clearly show that all the mutants have a significant cold acclimation defect. We opted to use the freezing test as the method to show a biological effect in our mutants since it is well described, widely accepted in the field and is a robust technique to test cold acclimation. Thus, in our opinion, the freezing test is optimal and enough to describe the biological role in the context of questions in the study and transient cold stress response. Survival rates in mutants for both sense-antisense, in the temperature range of 4°C to 16° C, can add further insights into biological effect, in line with performed freezing test. Our focus in this manuscript is to provide a mechanistic rationale and approach for addressing the cold induced transient transcriptional interplay between sense-antisense phenomenon and whether this widespread antisense transcription regulates sense gene's transcriptional reprogramming during early cold stress.***

Minor points:
1. Line 30: plaNET-seq was not introduced in the Abstract.

Thank you for noticing this mistake. The abbreviation has now been spelled out.

2. Line 38: the meaning of "positive role" here is obscure.

We have changed the wording to "positive regulatory role" in the manuscript.

3. Figure 1B-C: can the authors clarify whether the GO terms shown here were in the top list or handpicked? It would be better if the authors showed all the enriched GO terms in a supplemental table.

The GO-terms were the top 8 in the list. We have added the full list as supplemental tables (Supplemental Data Set S1).

4. Figure 1D: please indicate sense and antisense in the figure.

Done.

5. Line 127: please define "short genes".

We have changed the phrasing to: Overall, PAS genes tended to be shorter than other expressed genes.

6. Figure 2: "PAS host genes" were compared with "Non-PAS genes" in figure 2; however, it was described in the text and figure legends that PAS genes were compared with all expressed genes (e.g., line 129-130). Please clarify.

This has been clarified in the new figure 2.

7. Line 143: please also define DE here (FDR, fold change, ...)

We have added fold change to the text.

8. Line 156: define "responded".

We have changed the phrasing to "changed their expression significantly".

9. Line 230: "...283 bp deletion 331 bp..." the numbers here didn't match with what was shown in Figure 8A. Please clarify.

This has been clarified in the new Figure 9.

Reviewer #2 (Comments for the Author):

Work by Shiv Kumar Meena et al. aims to use plaNETseq to uncover antisense transcription in response to cold. They then select two examples and characterise the antisense and sense transcripts.
In my view, this manuscript requires a relatively high amount of work before it is mature.

1. I have a problem with the selected genes ZAT5 and BBX28. In the text, the authors say they selected them because the genes "responded positively to cold temperatures (3h of 4{degree sign}C) in the plaNET-seq data but not in the RNA-seq data (Supplemental Data Set S5)" but then Fig 5A and B show that those genes' steady-state RNA level is responsive to cold at similar data points as used for RNAseq?

We agree that our initial manuscript did not explain the selection of ZAT5 and BBX28 well. To clarify this point, we have made a several changes to our resubmitted manuscript. Our interest was mainly in the transcription factors that responded to cold temperatures. After the initial characterization (Fig. 1), we have now divided PAS genes into TFs and non-TFs (Fig. 2). From these cold responsive TFs, we selected those that had a similar expression profile of a known cold-responsive TF (CBF2, Fig. 3). 25 TFs fitted these conditions and we selected 5 of these genes for further analysis. These genes were chosen by their plaNET-seq expression profile. We have explained this better in the text, from line 172 and forward. We extensively attempted to generate CRIPSR lines for all five genes used in Figure 4 but were only able to get lines for* ZAT5 *and* BBX28 *with shorter deletions around antisense TSS to largely preserve the genomic context, owing to limitations posed by gRNAs, unwanted deletions, off/targets etc. This criterion could be fulfilled by CRIPSR lines for* ZAT5 *and* BBX28 *and thus we proceeded with* BBX28 *and* ZAT5*. On the other hand,

as part of this manuscript, we could characterize the novel cold stress role of these transcription factors. Reviewer's comment regarding Fig. 5a and 5b (now figure 7a and b) have been clarified in text.

2. If the authors are right and the BBX28 and ZAT5 nascent RNA is induced by cold but steady-state RNA is not then how can they control the phenotype? Maybe it is at later time points that the mRNA is affected? If it is not the mRNA that has the phenotypic effect then a trans-target for nascent RNA? This is very mysterious and needs to be clarified.

Thank you for your comment. The data in our resubmitted manuscript clearly shows that both the mRNA for* BBX28 *and ZAT5 have a decreased stability at 4°C compared to 22°C. Therefore, to keep up the steady state levels, both genes require increased transcriptional activity (shown by the plaNET-seq data in our manuscript). This suggests that the plant needs to keep a similar mRNA level of* ZAT5 *and* BBX28 *to cope with cold temperatures. If the plant does not keep the same nascent transcription of the genes, they will be downregulated due to their rapid mRNA turnover. That, in turn, could trigger a cold sensitive phenotype, as shown in freezing tests for antisense KO lines of BBX28 and ZAT5 (figure 9). At the same time, this highlights the advantage of using plaNET-seq during stress response, these genes would be challenging to detect with steady state methods. With our new RNA-seq analysis,* BBX28 *does have a significant induction after 3h and both* BBX28 *and* ZAT5 *show a tendency to be upregulated although it is not significant in all experiments. Further, to address reviewer's concern, we performed qPCR at later time points (24h and 48h) and mRNAs levels for BBX28 and ZAT5 stayed similar (Figure 7) supporting our results that early transcriptional programming is important for priming cold acclimation. The possibility of other trans target(s) for nascent RNA cannot be completely ruled out. However, our new results (Fig. S4, reporter LUC constructs with and without antisense transcription for ZAT5) reinforce our previous conclusion that sense expression is positively regulated by its antisense transcript during cold.

3. The CRISPR-generated lines are important but:
a. they are not well characterised. At least: nascent RNA (chromatin attached) RNA level quantification of sense and sense RNA stability are needed.

We agree and thank the reviewer. We have added several experiments to characterize the CRISPR lines better. Nascent RNA levels have been added to Fig. 9 for both 22°C and 3h at 4°C. Further characterizing the lines as per the reviewer's suggestion, we have also added mRNA stability of BBX28 and ZAT5 in the CRISPR lines (Fig. S5). These experiments show that the level of transcribing RNAPII is lower for both sense and antisense in the CRISPR mutants and that the mRNA stability is not changed.

b. More importantly a CRISPR deletion of a region behind a gene can have an effect on termination of the sense gene irrespective of how strong the authors stress it is not the case. So a couple of more approaches are required, like transgenes with 3' end of the sense gene exchanged for NOS or Rubisco terminators.

We thank the reviewer for this insightful comment. In fact, this was one of our main concerns why we progressed with the choice of BBX28 and ZAT5 as far as CRISPR lines are concerned. In the resubmitted manuscript and considering the reviewer's suggestion, we have added Supplemental Figure 4 to address the concerns. We generated two* LUCIFERASE *fusion constructs for ZAT5 by GreenGate cloning approach, one with the endogenous DNA sequence (including sense and antisense promoter), the other one with the NOS terminator replacing the 3'-UTR and antisense

promoter of ZAT5. The results show a general lower expression of* ZAT5 *in the tNOS construct and a decreased responsiveness to cold. These results further corroborate our earlier conclusions that the antisense transcript is important for proper expression of the sense gene during cold stress. Furthermore, the mRNA stability (Fig. S5) was similar between wild type and CRISPR lines, which rationalizes CRISPR lines data and argues for a limited effect of the deletions on sense termination.

4. An important technical aspect is the author's ability to measure the antisense. How sure are they that what they see in qPCR is not the sense transcript? One way to distinguish would be to measure the antisense level in sense t-DNA lines. If sense (behind T-DNA) is down but antisense is not affected, they are good. If antisense is affected, then of course it may be a biological effect of sense transcription on the antisense promoter firing.

Thank you for your comment. All cDNA synthesis for qPCR reactions were performed with a tagged oligo that included a sequence which is not found in Arabidopsis. This sequence was later used in the qPCR reaction to ensure strand specificity for sense and antisense and to avoid any genomic DNA amplification. This approach has been used extensively in previous papers for strand specific amplification (e.g., PMID: 36681861, PMID: 27856735). In addition, if we only amplified the sense transcript, we would expect to detect the same trends for sense and antisense transcripts. This is not the case, see Figure 9, where sense and antisense transcripts show distinct expression trends.

I also think the authors missed several important papers:

Immediate transcriptional responses of Arabidopsis leaves to heat shock (10.1111/jipb.12990) a paper describing nacent RNA versus stabel RNA in response to heat. So very similar to what the authors describe. "Direct measurement of transcription rates reveals multiple mechanisms for configuration of the Arabidopsis ambient temperature response" - From PubMed Central: PMC4053849, a paper that looks at the heat effect on RNA stability and transcription. Reaching a conceptually similar conclusion about buffering of the transcription effects by RNA stability.

We thank the reviewer for pointing these important papers out. We have added these references in the discussion.

"Arabidopsis SWI/SNF chromatin remodeling complex binds both promoters and terminators to regulate gene expression". Pubmed ID: PAMID 27994035
A paper describing a collection of genes with antisense transcription. Would be nice to compare the set of genes the authors described with the one described in this manuscript.

Thank you for the comment and highlighting crucial paper that we previously also considered during our study. The mentioned paper used microarray analysis to detect sense and antisense transcripts that are potentially regulated by the SWI/SNF subunit BRM. Addressing reviewer's concern, compared to the plaNET-seq data, we found 625 common genes between the BRM dataset and our PAS genes. 1133 genes were unique to the BRM dataset and 2512 unique to plaNET-seq defined PAS genes. We have added this paper to the discussion as well.

Minor points:

#90 "Nevertheless, lncRNAs have been characterized to control "
lncRNA should be changed to antisense RNA or similar.

We have changed lncRNA to antisense RNA.

#108 "Importantly, experimental validation demonstrated that their antisense transcripts are positively correlated to the stress responsiveness of the sense transcription, indicating a general role for antisense " Well experimental validation of 2 examples (as suggested by the previous sentence) can not indicate a general role for antisense. I recommend tuning down and saying something like: "indicated a possibility of" or "suggested that".

We have toned down this sentence.

Reviewer #3 (Comments for the Author):

In this manuscript, Meena and colleagues seek to characterize a class of antisense RNAs previously defined based on overlap with the sense gene's polyadenylation site, PAS-associated antisense RNAs. I certainly agree that up to this point it has been difficult to generalize what functional roles antisense RNAs may play as a class, if generalizations are possible. The motivations of this study are warranted and I am happy to see work pointing in this direction. However, substantial changes and additions to this manuscript would be necessary to support the central claims.

My largest concerns revolve around a lack of clarity and transparency about the datasets that were generated and/or used in this study. Additionally, the gene editing experiments performed to test whether antisense transcription affects sense gene products do not rule out a direct impact on sense transcription.

Major questions:
1. Key pieces of information are missing that prevent me from assessing the correctness of the work. The GitHub page on line 448 (https://github.com/martiquevedo/Kumar_etal_2022) is an empty repository. No details are given about the RNA-seq libraries generated for this study. How many replicates were made? How deeply sequenced were the libraries? Without any technical information about the quality and content of these libraries, I cannot rule out that the lower number of DEGs in RNA-seq vs. plaNET-seq in this case is simply a statistical artifact from, for example, poor within-treatment correlation.

We have added information about the RNA-seq experiment to the methods and additional information has been added to the GitHub and GEO depositories. RNA-seq was done with 3 biological replicates and sequenced to a similar depth to plaNET-seq (40-60 million raw reads for RNA-seq versus 50-100 million raw reads for plaNET-seq).

2. Supplemental Data Set S3 reports 1441 upregulated DEGs and 1584 down at 3 hours from RNA-seq, and 2274/2509 up/down at 12 hours. These gene sets are nearly twice as large as what is depicted in the white+dark gray bars of Fig3A. Unless I am missing something, these two pieces of data should correspond?

This was a mistake on our side that has been rectified so the numbers correspond. We have also rerun our RNA-seq analysis with an updated pipeline, so the numbers are different from our initial submission. Thank you for pointing this out.

3. The manuscript shows results from a number of public sequencing datasets without providing a proper "chain of custody". I assume that the plaNET-seq datasets referenced in Figure 1D are the same as those on GEO under accession GSE131733. But there are two reps for each timepoint there and only one track each in Fig1D, Fig4A-B, Fig7A-B, and FigS1. Were the reads pooled? If so, how?

For Fig7, where did the CAGE and DRS-seq data come from? Are the tracks labeled "CAGE" the same as the Nielsen et al. 2019 [1] TSS-seq data mentioned on line 451? I found no source for the DRS-seq data in the methods or references.

We agree that this was not explained well in our previous submission. This has been rectified in the methods of our resubmitted version. In all cases, raw data was collected from the source and processed as described in respective paper. All replicates have been merged and shown in the screenshots in our manuscript. We have put all references for the genome-wide data in the method section.

4. (Fig 2D) The tendency to find PAS-associated asRNA at highly transcribed genes could be interpreted as a consequence of extraneous polII binding due to high local polII occupancy.

We believe that this might be a misunderstanding. We do not see antisense transcription only at highly expressed genes. There is a large variation in the nascent transcription of PAS genes which would argue against this point.

Across all PAS genes, are changes to asRNA transcription observed before or after changes to the sense RNA? Is the time resolution of the previous plaNET-seq experiments sufficient to determine this?

We have included the new Fig. S2 to address this concern. The new figure shows PAS expression at the host genes that respond positively to cold temperature after 3h at 4°C (i.e., it includes our candidate genes **BBX28** ***and*** **ZAT5*****). There is no clear correlation between PAS expression and host gene expression, arguing against a direct regulatory role for PAS during the early cold response. Furthermore, the changes to nascent transcription level of the PAS compared to the host gene for this set of PAS genes are relatively small compared to the sense expression level. This point is exemplified well by our two candidate genes where small differences up and down for the PAS is contrasted by massive induction of the sense expression after 3h at 4°C. Although we cannot fully rule out that we might miss some general trends in PAS expression due to resolution issues, we find it unlikely since the first wave of transcription regulation occurring during cold exposure normally peaks after around 3h (i.e., the CBFs and other important transcription factors have their expression maxima). Thus, any important trends should be visible after 3h.***

5. The gene editing experiment designed to test the effects of asZAT5 and asBBX28 cannot rule out a direct effect on sense gene transcription. Some of the earliest arabidopsis transgenics showed that sequences downstream of a gene can be essential for its expression [2]. Moreover, the plaNET-seq data in Fig4B shows that sense transcription robustly extends beyond the BBX28 PAS by ~500bp. I do not see how a large deletion 193bp downstream "exclude<s> any potential effect on sense transcription" (line 243). To assess a causal relationship between the sense and antisense RNAs would require inducible activation or knockdown of the asRNA, such as with a pER8 promoter construct as shown by Zhao et al. 2018 [3].

We appreciate this comment which made us explain this part better in the resubmitted manuscript. First, we want to make it clear that the GL1 gene, investigated by Larkin et al has an antisense transcript (Fig. 1, below). The enhancer-like sequence identified in the paper corresponds to the sequence close to the TSS of the antisense transcript. The T-DNA line used in the paper has a similar insertion site to many T-DNA lines used in antisense transcript characterization (PMID: 36681861, PMID: 36857348). Thus, many "enhancer" regions downstream of genes could be the consequence of antisense transcription. Sense transcription beyond the PAS (read-through

transcription) is highly unstable and disruption of it is not believed to affect termination of the sense mRNA (PMID: 30385760).

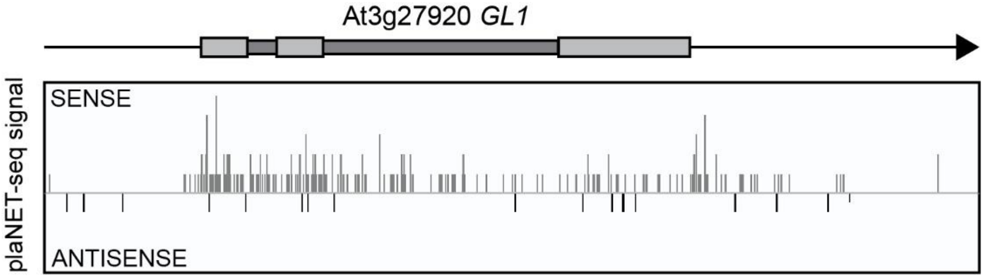

Figure 1. Screenshot of the *GL1* gene.

To further address this point, we have included nascent data and mRNA stability data in the CRISPR lines. The stability data show that we do not affect the termination of the mRNA in the CRISPR lines. Considering reviewers' suggestions to address sense-antisense relationship using transgenic constructs, we have also added results from transgenic constructs (Fig. S4) that support our earlier conclusions.

The amiRNA approach used by Zhao et al requires a stable RNA that the amiRNA can target. It could be a good alternative to downregulate antisense transcript in stand specific manner but has limitations such as e.g. cytosolic localization of target transcript, amiRNA processing to mature form during stresses such as cold etc. Therefore, universal applicability of amiRNA approach is not yet established. Nevertheless, in our case, both cold induced antisense transcripts are rapidly degraded by the nuclear exosome, making it unsuitable to be targeted by amiRNAs. Thus, the amiRNA approach is ill suited in our case to downregulate the antisense transcripts.

We have changed the wording of line 243 (now line 297) to "the direct interference with sense transcription would be minimized" since we cannot rule out this completely. Nevertheless, we believe our new results strongly suggest that we only directly affect antisense transcription in our CRISPR lines.

6. After emphasizing that measuring steady-state RNA is unsuitable for studying the early cold stress response, the effects of the two CRISPR mutant lines are quantified by measuring steady-state RNA at single time point (Fig8B-C,F-G). Both asZAT5 and asBBX28 are still detected in their respective mutant lines; are their nascent transcripts still cold-responsive in the same was as shown in FigS2? Measuring nascent sense and antisense transcription over a time course of cold treatment in wild-type and the two CRISPR lines would be necessary to show (1) whether cold-induced changes are affected by the deletions and (2) if changes to antisense RNA transcription precede changes to sense RNA transcription. The arrow of causality cannot be determined by the results shown.

We have addressed this thoughtful comment with new experiments. The reviewer is correct, showing steady state levels of the antisense transcript can lead to false conclusions. Therefore, we have removed all steady state measurements of PAS in our resubmitted manuscript. Our new Figure 9 includes both nascent and steady state levels of sense transcripts and nascent transcription for the PAS at two time points. Both CRISPR lines show a decreased antisense transcription at 22°C and a severely mis-regulated sense transcription after cold treatment. It is not surprising that we still detect antisense transcription in the CRISPR mutants since these are not full knock out mutants, we only remove parts of the antisense promoter. What is clear is that both mutants are unable to respond to cold stress. Both BBX28 and ZAT5 mRNA levels in respective mutant are similar between 22°C and 3h 4°C.

Other points:
1. (line 155) What percent of non-PAS genes respond to cold at one or both time points? Are PAS genes responsive to a degree that other genes are not?

An overwhelming majority of PAS genes were responsive to cold by either RNA-seq, plaNET-seq or both (86%), corroborating the enriched GO-terms. This has been added to the results.

2. (line 178) Even if it is not statistically significant, the 2 hour timepoint measurements in Fig5A are nearly 2-fold higher than at 22C, and the directions of change agree with the plaNET-seq fairly well. Measuring nascent and mature RNA simultaneously during the same time course would give more valuable insights here.

This has been done in the new Figure 9.

3. (line 184) A brief description of the electrolyte leakage assay here would be important to improve readability, along with an explanation of what is meant by "freezing tolerance" (i.e., LT50).

We have explained this better in the text (line 247 and forward).

4. (line 215) Here it is not made obvious that, while both asRNAs show differences after cold, the changes are opposite to each other (asZAT5 up and asBBX28 down, FigS2A-B). How can this be reconciled with a model where both asRNAs increase sense transcription?

Thank you for this comment. We argue in the manuscript that antisense transcription is important for priming the stress responsiveness of the host gene. To strengthen our model, we have added Fig. S2 that shows that PAS expression for PAS host genes upregulated after 3h at 4° only changes marginally compared to the sense expression (Figure 4). The low level of antisense transcription compared to sense transcription is therefore unlikely to have a strong regulatory effect during the initial stages of cold response. We agree that changes to asZAT5 and asBBX28 expression in response to cold is the opposite, but these changes are miniscule to the changes to sense expression and exemplify our point well. A more likely explanation and therefore integrated into our model is that constant but low antisense transcription during control conditions primes the host gene for stress response. The nascent RNA analysis in the new Figure 9 also corroborates this conclusion. Both sense and PAS expression are affected in the CRISPR mutants already at 22° which affects the stress responsiveness of the host genes.

5. (line 411) The URL provided gives a 404 error.

This has been fixed. We have added a reference to the methods.

6. Was the difference between nascent RNA and mature RNA abundance during the cold response not already shown in Kindgren et al. 2019 [4] by comparing plaNET-seq to TSS-seq (Fig4C)?

Thank you for your comment. In the previous study we only provided a global view. In the current study, we have not only expanded time frame (12h 4°C) but also attempted to underscore a mechanistic basis by functionally characterizing PAS host genes to draw attention of global RNA community for exploring the phenomenon of regulatory antisense transcription in plants. RNA-seq is also good since it measures RNA levels across the gene body, while plaNET-seq has an additional upper edge particularly for short lived transcripts. TSS-seq on the other hand, measures only initiated transcripts, which could result in discrepancies when comparing with plaNET-seq. We think thorough plaNET-seq Vs RNA-Seq comparison in present study enabled us to explore starting point of regulatory PAS antisense transcription both methodologically and conceptually.

_____

1. Nielsen M, Ard R, Leng X, Ivanov M, Kindgren P, Pelechano V, et al. Transcription-driven chromatin repression of Intragenic transcription start sites. PLOS Genetics. Public Library of Science; 2019;15:e1007969.

2. Larkin JC, Oppenheimer DG, Pollock S, Marks MD. Arabidopsis GLABROUS1 Gene Requires Downstream Sequences for Function. The Plant Cell. 1993;5:1739-48.

3. Zhao X, Li J, Lian B, Gu H, Li Y, Qi Y. Global identification of Arabidopsis lncRNAs reveals the regulation of MAF4 by a natural antisense RNA. Nat Commun. Nature Publishing Group; 2018;9:5056.

4. Kindgren P, Ivanov M, Marquardt S. Native elongation transcript sequencing reveals temperature dependent dynamics of nascent RNAPII transcription in Arabidopsis. Nucleic Acids Research. 2020;48:2332-47.

_____

Dr. Peter Kindgren
Umea Plant Science Centre
Forest Genetics and Plant Physiology
Umeå
Sweden

Dear Dr. Kindgren:

We have received reviews of your manuscript entitled "Genes hosting antisense transcripts are stress responsive and govern cold acclimation in Arabidopsis thaliana." Thank you for submitting your best work to The Plant Cell. The editorial board agrees that the work you describe is substantive, falls within the scope of the journal, and may become acceptable for publication, pending revision and potential re-review.

In addition to addressing each of the reviewers' points, it is critical that overall the revision solidifies the connection to antisense transcription, as multiple reviewers raised the issue that this aspect of the manuscript is not fully convincing.

TPC is reluctant to see manuscripts undergoing multiple rounds of revision, so it is very important to ensure that the upcoming revision satisfies reviewers in every reasonable aspect of the queries and constructive criticisms put forward.

Please contact us if there are ambiguous comments or if you wish to discuss the revision.

Given the nature of the comments, we are offering you 60 days from when we have issued this decision to complete the revision. If a revision is not returned within this time frame, and if you have not been granted an extension, we will withdraw the manuscript, which will leave you free to submit the work elsewhere. If you need an extension, we encourage you to contact us at any point before the 60 days have passed.

When you are ready to submit the revised version, please upload a highlighted copy that indicates all changes made in response to the editor and reviewer recommendations. Include an itemized list of all changes made in response to each of the reviewer's suggestions in the "Response to Reviewers" section; please note that reviewers do not have access to your cover letter, nor was this decision letter shared with them.

Thank you for the privilege of reviewing your work. We look forward to receiving your revised manuscript.

Sincerely,

The Plant Cell Editorial Board

Please note the following:

-The Plant Cell now requires authors to complete and submit an author revisions checklist upon submission of a revised manuscript. The aim of the checklist is to aid authors in preparing a high-quality manuscript, facilitate the review and assessment of revised manuscripts, and help to ensure that journal standards are maintained across the board. If your manuscript is accepted, the completed checklist will be published as supplemental material attached to the article online. Please download a copy of the checklist (pdf fillable form) at this link, for submission with your revised manuscript: https://tpc.msubmit.net/html/Author_Revisions_Checklist.pdf.

-Supplemental materials should be restricted to large datasets and tables, presentation of replicates, and validation of reagents, methods, or genotypes. Any data that are used to support the major claims must be in the main manuscript. Supplemental figure legends must indicate what figure in the main manuscript is supported by the supplemental data presented. Please justify how each of the supplemental figures meet the criteria.

-Sampling methods and nature of "biological replicates" should be described precisely (i.e. different plants, parts of plants, pooled tissue, independent pools of tissue, sampled at different times, etc), along with a clear description of and rationale for any statistical analyses conducted. The reader should know exactly what was sampled; what forms the basis of the calculation of any means and statistical parameters reported. This is also necessary to ensure that proper statistical analysis was conducted.

-Want to add this revision deadline to your calendar? Click below!

-- Reviewer comments:

Reviewer #1 (Comments for the Author):

The authors have addressed all of the queries I raised and have implemented necessary modifications to the manuscript. While I concur with the primary conclusions drawn in this manuscript, I believe a few adjustments or clarifications are essential before considering acceptance.

1. Page 2, Background: The term "non-coding" is used, encompassing DNA encoding rRNAs, tRNAs, miRNAs, snRNA, snoRNAs, etc. However, it may be more precise to specify DNA for long non-coding RNAs. Please refine this statement for accuracy.

2. I suggest the authors adopt a more neutral tone when presenting their findings. While I acknowledge the potential of plaNET-seq in identifying novel stress response genes, it's imperative to address and elucidate its limitations in the manuscript. Notably, transient enhancements in gene expression may not suffice for a gene product to significantly contribute to stress response; sustained, albeit not always robust, expression could be critical, particularly for genes coordinating with the translation machinery. Hence, both plaNET-seq and RNA-seq methodologies hold promise in identifying pivotal genes. Upon reviewing Figure 3A&B, it appears that quite a few genes were only differentially expressed in RNA-seq data, not in plaNET-seq. I'm wondering whether any well-known cold stress-related genes exhibited this pattern. If observed, discussing the limitations of plaNET-seq in capturing such genes becomes essential.

3. Figure 1D Legends: The legend should state "3 hours" instead of "4 hours."

4. Lines 196-197: Correct the grammar mistake for improved clarity.

5. Figure 3: For all R2 values, replace "," with ".".

Reviewer #2 (Comments for the Author):

This is a revised manuscript from Shiv Kumar Meena et. al. The authors have improved several aspects of the manuscript, providing transgene analysis for antisense function.

I however still feel this is a very preliminary report with many loose ends and no coherent message. I also feel some of the points raised by me have not been addressed - see below.

The lack of coherent message is reflected in the title, in the current version: "Genes hosting antisense transcripts are stress responsive and govern cold acclimation in Arabidopsis thaliana."

That genes with antisense are stress-responsive is well known, and that they govern cold acclimation is a bit tricky as the authors tested two genes only.

Apart from this, I guess the main conclusions are:
- the link of antisense to RNA stability
- the "active transcription" changes and biological effect
- the activating role of antisense in the case of the two examples studied.

As for the link to RNA stability, authors failed to detect changes in RNA stability when the selected antisense was manipulated. So this is based only on the correlation shown in Fig 2C. Here the authors show that TF genes with antisense transcripts have a much lower stability than all genes or genes with antisense that are not TF. Now this is a bit unfair comparison as the TF with AS should be compared to TF without AS and not to all genes. As for now, data suggest that it is NOT the AS per see that results in lower stability of these genes.

Somehow linked to the previous point the authors conclude that:
"Changes in the active transcription of a gene measured by plaNET-seq have biological relevance, even though the steady-state level of the gene's mRNA is similar to control conditions after cold exposure (Figure 4-5). "

This is a point I raised in my previous version of the review and I do not feel it has been addressed properly. It could be formally possible that antisense transcription results in high turnover and as a result despite the observed lack of effect on steady-state RNA level changes observed using netSEQ are relevant for the phenotype. However, as described above and acknowledged by the author in the discussion their experimental data do not support the role of antisense in RNA stability control.

Another possibility could simply be that active transcription measured by netSEQ manifests itself at a stable RNA level later on, and this is translated into the phenotype. But this is quite obvious, and in my opinion, is not reflected in the statement: "Changes in the active transcription of a gene measured by plaNET-seq have biological relevance, even though the steady-state level of the gene's mRNA is similar to control conditions after cold exposure (Figure 4-5). " any many similar throughout the manuscript.

Finally, the manuscript contains data that suggest an activating role of antisense. Here the data looks quite convincing but in my view is preliminary. The model assumes that AS is downregulated by cold and as a result, sense transcription is induced. That could be true but requires analysis of the separated sense and antisense promoter-reporter fusions for conclusive evidence.

Reviewer #3 (Comments for the Author):

In this resubmitted manuscript, Meena and colleagues have addressed a number of concerns raised during the first round of review. The updated manuscript has substantially improved documentation of data sources, code, and analysis methods. They have also performed additional experiments to better characterize the CRISPR mutant lines and the effect of the 3' region of ZAT5 on protein abundance. Most of my initial concerns have been adequately addressed, and I think the overall ideas presented in the manuscript are worthwhile. However, I still have questions about the relevance of antisense transcription in the two selected genes, ZAT5 and BBX28.

In both the RNA-seq and plaNET-seq, differential expression is calculated vs. the 22{degree sign}C 0hr timepoint, meaning DEGs are a product of both changing temperature and changing time. Both ZAT5 and BBX28 are reported as circadian-regulated genes (see e.g. Romanowski 2020 Table S3, https://doi.org/10.1111/tpj.14776), so their expression is expected to change over a 12-hour timecourse regardless of temperature. Have the authors performed any experiments that disentangle temperature effects and circadian effects? For example, comparing to "mock" 3hr and 12hr plants kept at 22{degree sign}C?

Some new results in Figure 9 conflict with other earlier sections in the paper:
[Line 241] states that "the steady-state level of ZAT5 was not significantly increased" in contrast with plaNET-seq results, and this is explained by an increase in ZAT5 turnover in cold (Fig5D). But the new measurements in Fig9B show a larger fold-increase of steady-state ZAT5 than nascent ZAT5 after 3 hours at 4{degree sign}C. How can this observation be reconciled with the other results?
[Line 237] The question "Can mRNAs with equal steady state levels during stress have an important biological role in the stress adaptation for the plant?" is not answered by the examples of ZAT5 or BBX28, because their steady state levels do not stay unchanged.

Line 147-150: Examining transcription factors as a separate group is an interesting idea, but Sorenson et al. observed that TFs generally have a higher turnover rate than non-TF genes. Whether PAS transcription specifically is associated with a higher turnover rate of transcription factor RNA would be better demonstrated by comparing "PAS TFs" to "non-PAS TFs", instead of to "PAS non-TFs"

Minor points:
The most well-studied cold responsive antisense RNA in Arabidopsis is COOLAIR (AT5G01675). It matches the definition of PAS gene defined here, so I am surprised not to see FLC (AT5G10140) listed in the set of PAS host genes in Supplemental Dataset 1. Could the authors clarify why FLC/COOLAIR was not identified in this study?

Throughout the manuscript the authors refer to "genes hosting antisense transcripts"; because these are nascent polII products that aren't detected in the steady state pool, would the more accurate phrase be "genes hosting antisense transcription"?

Line 50 could be reworded to "What importance does PAS transcription have in the cold response?"

Reviewer #1 (Comments for the Author):

The authors have addressed all of the queries I raised and have implemented necessary modifications to the manuscript. While I concur with the primary conclusions drawn in this manuscript, I believe a few adjustments or clarifications are essential before considering acceptance.

1. Page 2, Background: The term "non-coding" is used, encompassing DNA encoding rRNAs, tRNAs, miRNAs, snRNA, snoRNAs, etc. However, it may be more precise to specify DNA for long non-coding RNAs. Please refine this statement for accuracy.

The statement is now refined to long non-coding.

2. I suggest the authors adopt a more neutral tone when presenting their findings. While I acknowledge the potential of plaNET-seq in identifying novel stress response genes, it's imperative to address and elucidate its limitations in the manuscript. Notably, transient enhancements in gene expression may not suffice for a gene product to significantly contribute to stress response; sustained, albeit not always robust, expression could be critical, particularly for genes coordinating with the translation machinery. Hence, both plaNET-seq and RNA-seq methodologies hold promise in identifying pivotal genes. Upon reviewing Figure 3A&B, it appears that quite a few genes were only differentially expressed in RNA-seq data, not in plaNET-seq. I'm wondering whether any well-known cold stress-related genes exhibited this pattern. If observed, discussing the limitations of plaNET-seq in capturing such genes becomes essential.

We thank the reviewer for this comment and agree. We do not propose that plaNET-seq is better than RNA-seq to detect changes to the transcriptional output following cold treatment. Rather, both methods combined complement our understanding of how transcription adapts to colder temperatures. Any notion of a different view was unintentional from our side. We have tried to edit this to provide a comprehensive and balanced message in the manuscript. It is important to note, however, that plaNET-seq has the advantage over RNA-seq to detect significant differences in mRNAs with high dynamic regulation (high transcription activity and high mRNA turnover).

The differentially expressed genes unique to the RNA-seq analysis were not enriched in "cold responsive" or "temperature responsive" genes. Looking through the gene list, we could not identify any well characterized cold stress-related genes.

3. Figure 1D Legends: The legend should state "3 hours" instead of "4 hours."

Thank you for pointing out. We have changed the legend as stated by the reviewer.

4. Lines 196-197: Correct the grammar mistake for improved clarity.

We have rephrased this part of the manuscript.

5. Figure 3: For all R2 values, replace "," with ".".

Fixed.

Reviewer #2 (Comments for the Author):

This is a revised manuscript from Shiv Kumar Meena et. al. The authors have improved several aspects of the manuscript, providing transgene analysis for antisense function.

I however still feel this is a very preliminary report with many loose ends and no coherent message. I also feel some of the points raised by me have not been addressed - see below.

The lack of coherent message is reflected in the title, in the current version: "Genes hosting antisense transcripts are stress responsive and govern cold acclimation in Arabidopsis thaliana." That genes with antisense are stress-responsive is well known, and that they govern cold acclimation is a bit tricky as the authors tested two genes only.

We thank the reviewer for suggestion. We have changed the title to better encompass the message of the manuscript:

Antisense transcription from stress responsive transcription factors fine tunes the cold response in *Arabidopsis thaliana*: insights from *BBX28* and *ZAT5* characterization.

Apart from this, I guess the main conclusions are:

- the link of antisense to RNA stability

- the "active transcription" changes and biological effect

- the activating role of antisense in the case of the two examples studied.

We address the different points in detail below.

---

1. As for the link to RNA stability, authors failed to detect changes in RNA stability when the selected antisense was manipulated. So this is based only on the correlation shown in Fig 2C. Here the authors show that TF genes with antisense transcripts have a much lower stability than all genes or genes with antisense that are not TF. Now this is a bit unfair comparison as the TF with AS should be compared to TF without AS and not to all genes. As for now, data suggest that it is NOT the AS per see that results in lower stability of these genes.

We think there might be misunderstanding here, and we thank the reviewer to bring it up so we can explain our reasoning better to avoid confusion. We do not claim that antisense transcription is involved in the mRNA stability of the host gene. To the contrary, our results (Fig. S1, Fig. 2, Fig. S2, Fig. 5, Fig. S5, and Fig. 9) strongly suggest that antisense transcription is not linked to mRNA stability. We have thoroughly edited the text to avoid any speculated notion that antisense transcription might have a general role in determining mRNA stability.

We agree with the reviewer that a comparison with non-PAS TFs is useful to bring clarity to this concern. We have updated Figure 2 and Supplemental Figure 1 with new data including TFs without antisense transcription. The correlation is clear between the length of a gene and the presence of a PAS (Fig. 2A). The data in Figure 2B-D and Supplemental Figure 1 show that TFs in general have a high turnover rate (i.e. independent of antisense transcription). However, genes with a PAS tend to have higher transcription rate, PAS-TFs represent an extreme case of high transcription and rapid turnover at normal growing temperatures. Thus, PAS-TFs show a more dynamic regulation compared to other PAS genes and other TFs. The dynamic regulation of PAS-TFs is also evident during cold exposure (Fig. 5). Our main conclusion is therefore that a nascent RNA detection method (plaNET-seq) complements steady state methods (RNA-seq) that together notice a greater range of cold responsive genes.

2. Somehow linked to the previous point the authors conclude that: "Changes in the active transcription of a gene measured by plaNET-seq have biological relevance, even though the steady-state level of the gene's mRNA is similar to control conditions after cold exposure (Figure 4-5)”. This is a point I raised in my previous version of the review, and I do not feel it has been addressed properly. It could be formally possible that antisense transcription results in high turnover and as a result despite the observed lack of effect on steady-state RNA level changes observed using netSEQ are relevant for the phenotype. However, as described above and acknowledged by the author in the discussion their experimental data do not support the role of antisense in RNA stability control.

Thank you for this comment. Please, see the answer to the previous point where we point out that nothing in our results and previously published reports suggest a general role for antisense transcription in mRNA turnover. Our main conclusion here is that plaNET-seq, in contrast to RNA-seq, can detect those genes with dynamic mRNA regulation, both under normal growing conditions and after exposure to stress. Highly dynamic regulated genes include PAS genes that have high active transcription as exemplified in this manuscript. Furthermore, we have changed the statement identified by the reviewer and similar in the text to get a more balanced manuscript since we detect small but significant increases of mRNA levels of **BBX28** ***and*** **ZAT5** ***at certain time points and experiments. Hence, throughout the manuscript we avoid stating “equal” or “no” difference (in terms of mRNA level) and state minor differences instead.***

3. Another possibility could simply be that active transcription measured by netSEQ manifests itself at a stable RNA level later on, and this is translated into the phenotype. But this is quite obvious, and in my opinion, is not reflected in the statement: "Changes in the active transcription of a gene measured by plaNET-seq have biological relevance, even though the steady-state level of the gene's mRNA is similar to control conditions after cold exposure (Figure 4-5). " any many similar throughout the manuscript.

We agree with the reviewer that we cannot rule out an increase of mRNA later in the cold response. However, our focus in this manuscript is to identify means and provide a base to study the transient transcriptional response in the context of antisense transcription during cold. In agreement with reviewer, we have downplayed/changed these statements in the text. We would like to stress that the main message is that highly responsive and dynamically regulated genes are challenging to detect with steady state methods because of the built-in constraints of the methods. The statistical significance of the small differences we detect in the steady state levels of **ZAT5** ***and*** **BBX28** ***are thus highly dependent on the variation between biological replicates, stress treatment, and circadian regulation (as pointed out by reviewer 3, see Supplemental Figure S3A-B). In contrast, a nascent detection method increases the probability of finding significance in transcriptional activity over a gene body since it is independent of the high mRNA turnover for certain mRNAs.***

4. Finally, the manuscript contains data that suggest an activating role of antisense. Here the data looks quite convincing but in my view is preliminary. The model assumes that AS is downregulated by cold and as a result, sense transcription is induced. That could be true but requires analysis of the separated sense and antisense promoter-reporter fusions for conclusive evidence.

Our data from the CRISPR lines (Fig. 9) and LUC-fusion constructs (Fig. S4) cohesively suggest an activating role for antisense transcription. LUC-fusion constructs with and without antisense transcription disentangled the cis regulatory aspect. The low expression level and high turnover for the antisense transcripts studied here clearly supports a cis role. The experiment suggested by the reviewer would determine trans roles for antisense transcripts which is not a focus of this manuscript. However, we agree with the reviewer that these results are preliminary but quite convincing, and we have therefore been very careful not to state this as a conclusion for a general

role in the results and commented on it in the discussion to get a balanced manuscript. We do conclude that a proper regulation of* ZAT5 *and* BBX28 *require antisense transcription (see Fig. S4, Fig. 9) but the exact mechanism(s), of how this regulation is achieved, we believe is outside the scope of this manuscript but an obvious future avenue of research.

Reviewer #3 (Comments for the Author):

In this resubmitted manuscript, Meena and colleagues have addressed a number of concerns raised during the first round of review. The updated manuscript has substantially improved documentation of data sources, code, and analysis methods. They have also performed additional experiments to better characterize the CRISPR mutant lines and the effect of the 3' region of ZAT5 on protein abundance. Most of my initial concerns have been adequately addressed, and I think the overall ideas presented in the manuscript are worthwhile. However, I still have questions about the relevance of antisense transcription in the two selected genes, ZAT5 and BBX28.

In both the RNA-seq and plaNET-seq, differential expression is calculated vs. the 22°C 0hr timepoint, meaning DEGs are a product of both changing temperature and changing time. Both ZAT5 and BBX28 are reported as circadian-regulated genes (see e.g. Romanowski 2020 Table S3, https://doi.org/10.1111/tpj.14776), so their expression is expected to change over a 12-hour time course regardless of temperature. Have the authors performed any experiments that disentangle temperature effects and circadian effects? For example, comparing to "mock" 3hr and 12hr plants kept at 22°C?

This is a great point and we have included new data in Supplemental Figure S3A-B. The new data show that* BBX28 *had a small but significant increase in cold conditions after 3h and 12h.* ZAT5 *showed a significant increase after 3h but not after 12h. We have also added the reference to the text.

Some new results in Figure 9 conflict with other earlier sections in the paper: [Line 241] states that "the steady-state level of ZAT5 was not significantly increased" in contrast with plaNET-seq results, and this is explained by an increase in ZAT5 turnover in cold (Fig5D). But the new measurements in Fig9B show a larger fold-increase of steady-state ZAT5 than nascent ZAT5 after 3 hours at 4°C. How can this observation be reconciled with the other results? [Line 237] The question "Can mRNAs with equal steady state levels during stress have an important biological role in the stress adaptation for the plant?" is not answered by the examples of ZAT5 or BBX28, because their steady state levels do not stay unchanged.

We thank the reviewer for this comment. Together with the previous comment, it allows us to reconcile our results into an enhanced manuscript. The time series in Figure 5D did not find a significant increase of* ZAT5 *after 2h and 4h of cold. In Figure 9, we have used 3h of cold treatment and we see a small but significant increase (also reflected in the new Supplemental Figure 3B). These results highlight the small differences in mRNA levels at different time points which lead to significant results in some experiments but not in others, largely depending on the variation between biological replicates.

We agree with the reviewer that the question posed on line 237 was incorrectly formulated. We have changed this to "Can mRNAs with small differences in steady state levels during stress have an important biological role in the stress adaptation for the plant?" as this better captures the main message of the manuscript. Furthermore, we have toned down the rest of the text concerning similar statements as the question on line 237 for a more balanced manuscript.

Line 147-150: Examining transcription factors as a separate group is an interesting idea, but Sorenson et al. observed that TFs generally have a higher turnover rate than non-TF genes. Whether PAS transcription specifically is associated with a higher turnover rate of transcription factor RNA would be better demonstrated by comparing "PAS TFs" to "non-PAS TFs", instead of to "PAS non-TFs"

Thank you for this comment and suggestion. See answer to point 1 from reviewer 2.

Minor points:

The most well-studied cold responsive antisense RNA in Arabidopsis is COOLAIR (AT5G01675). It matches the definition of PAS gene defined here, so I am surprised not to see FLC (AT5G10140) listed in the set of PAS host genes in Supplemental Dataset 1. Could the authors clarify why FLC/COOLAIR was not identified in this study?

A good point that needs clarification. The list of PAS genes used in this manuscript are genes without an annotated antisense transcript prior to the plaNET-seq analysis (described in Kindgren et al, 2020). Thus, genes like **FLC** ***and its antisense transcript*** **COOLAIR** ***and a few other well described and characterized antisense transcripts are not part of the list. We have clarified this in the text to avoid any confusion.***

Throughout the manuscript the authors refer to "genes hosting antisense transcripts"; because these are nascent polII products that aren't detected in the steady state pool, would the more accurate phrase be "genes hosting antisense transcription"?

Agreed. This has been changed in the manuscript.

Line 50 could be reworded to "What importance does PAS transcription have in the cold response?"

Fixed.

Dear Dr. Peter Kindgren:

We have received reviews of your manuscript entitled "Antisense transcription from stress responsive transcription factors fine tunes the cold response in Arabidopsis thaliana: insights from BBX28 and ZAT5 characterization.." On the basis of the advice received, the board of reviewing editors would like to accept your manuscript for publication in The Plant Cell. This acceptance is contingent on revision based on the comments of our reviewers. In particular, please consider the following:

We very much appreciate the quality revision that has addressed all reviewer concerns! The only issue that remains to be addressed is checking the possible technical issue pointed out by Reviewer #3 regarding integration of the new data-- it seems that the Supplemental Figure 3 legend was expanded by two figures, but the Supplemental Material in the submission still contains only the original two panels; it's possible that the wrong version of the Supplemental Material PDF was uploaded.

To submit your revised manuscript (in this case a Supplement re-upload if this turns out to be the culprit of the issue), please click:

Link Not Available

If you have any questions about the revision submission procedures, please contact the Editorial Office Staff (tpc-submissions@aspb.org). If you cannot return the revised manuscript within 30 days, please let us know. Otherwise, we will assume that you have elected not to revise the manuscript and withdraw it.

Thank you very much for the privilege of reviewing this work. I look forward to receiving the next version.

Sincerely,

The Plant Cell Editorial Board

Reviewer #1 (Comments for the Author):

The authors have successfully addressed all my questions and made changes in the revised manuscript accordingly. I have no other questions.

Reviewer #2 (Comments for the Author):

Authors have significantly tuned down the conclusions, changed the title and clarified some points where needed. I believe the manuscript is now ready to be published.

Reviewer #3 (Comments for the Author):

I thank the authors for addressing my questions from the previous round. The changes to the main text in response to all reviewers were helpful to clarify the messages of the manuscript, and it has improved overall.

Unfortunately, I think there is a technical issue integrating the new data: The Supplemental Figure 3 legend was expanded by two figures, but the Supplemental Material file I see in the submission portal still contains only the original two panels. Perhaps the wrong version of the Supplemental Material PDF was uploaded.

If Figure S3A-B are as described in the rebuttal letter and figure legend, then my concerns have been addressed.

Please also note the following:

-The Plant Cell now requires authors to complete and submit an author revisions checklist upon submission of a revised manuscript. The aim of the checklist is to aid authors in preparing a high-quality manuscript, facilitate the review and assessment of revised manuscripts, and help to ensure that journal standards are maintained across the board. If your manuscript is accepted, the completed checklist will be published as supplemental material attached to the article online. Please download a copy of the checklist (pdf fillable form) at this link, for submission with your revised manuscript: https://tpc.msubmit.net/html/Author_Revisions_Checklist.pdf.

-Supplemental materials should be restricted to large datasets and tables, presentation of replicates, and validation of reagents, methods, or genotypes. Any data that are used to support the major claims must be in the main manuscript. Supplemental figure legends must indicate what figure in the main manuscript is supported by the supplemental data presented. Please justify how each of the supplemental figures meet the criteria.

-Sampling methods and the nature of "biological replicates" should be described precisely (i.e. different plants, parts of plants, pooled tissue, independent pools of tissue, sampled at different times, etc.), along with a clear description of and rationale for any statistical analyses conducted. The reader should know exactly what was sampled; what forms the basis of the calculation of any means and statistical parameters reported. This is also necessary to ensure that proper statistical analysis was conducted.

Reviewer #1 (Comments for the Author):

The authors have addressed all of the queries I raised and have implemented necessary modifications to the manuscript. While I concur with the primary conclusions drawn in this manuscript, I believe a few adjustments or clarifications are essential before considering acceptance.

1. Page 2, Background: The term "non-coding" is used, encompassing DNA encoding rRNAs, tRNAs, miRNAs, snRNA, snoRNAs, etc. However, it may be more precise to specify DNA for long non-coding RNAs. Please refine this statement for accuracy.

The statement is now refined to long non-coding.

2. I suggest the authors adopt a more neutral tone when presenting their findings. While I acknowledge the potential of plaNET-seq in identifying novel stress response genes, it's imperative to address and elucidate its limitations in the manuscript. Notably, transient enhancements in gene expression may not suffice for a gene product to significantly contribute to stress response; sustained, albeit not always robust, expression could be critical, particularly for genes coordinating with the translation machinery. Hence, both plaNET-seq and RNA-seq methodologies hold promise in identifying pivotal genes. Upon reviewing Figure 3A&B, it appears that quite a few genes were only differentially expressed in RNA-seq data, not in plaNET-seq. I'm wondering whether any well-known cold stress-related genes exhibited this pattern. If observed, discussing the limitations of plaNET-seq in capturing such genes becomes essential.

We thank the reviewer for this comment and agree. We do not propose that plaNET-seq is better than RNA-seq to detect changes to the transcriptional output following cold treatment. Rather, both methods combined complement our understanding of how transcription adapts to colder temperatures. Any notion of a different view was unintentional from our side. We have tried to edit this to provide a comprehensive and balanced message in the manuscript. It is important to note, however, that plaNET-seq has the advantage over RNA-seq to detect significant differences in mRNAs with high dynamic regulation (high transcription activity and high mRNA turnover).

The differentially expressed genes unique to the RNA-seq analysis were not enriched in "cold responsive" or "temperature responsive" genes. Looking through the gene list, we could not identify any well characterized cold stress-related genes.

3. Figure 1D Legends: The legend should state "3 hours" instead of "4 hours."

Thank you for pointing out. We have changed the legend as stated by the reviewer.

4. Lines 196-197: Correct the grammar mistake for improved clarity.

We have rephrased this part of the manuscript.

5. Figure 3: For all R2 values, replace "," with ".".

Fixed.

Reviewer #2 (Comments for the Author):

This is a revised manuscript from Shiv Kumar Meena et. al. The authors have improved several aspects of the manuscript, providing transgene analysis for antisense function.

I however still feel this is a very preliminary report with many loose ends and no coherent message. I also feel some of the points raised by me have not been addressed - see below.

The lack of coherent message is reflected in the title, in the current version: "Genes hosting antisense transcripts are stress responsive and govern cold acclimation in Arabidopsis thaliana." That genes with antisense are stress-responsive is well known, and that they govern cold acclimation is a bit tricky as the authors tested two genes only.

We thank the reviewer for suggestion. We have changed the title to better encompass the message of the manuscript:

Antisense transcription from stress responsive transcription factors fine tunes the cold response in *Arabidopsis thaliana*: insights from *BBX28* and *ZAT5* characterization.

Apart from this, I guess the main conclusions are:

- the link of antisense to RNA stability

- the "active transcription" changes and biological effect

- the activating role of antisense in the case of the two examples studied.

We address the different points in detail below.

---

1. As for the link to RNA stability, authors failed to detect changes in RNA stability when the selected antisense was manipulated. So this is based only on the correlation shown in Fig 2C. Here the authors show that TF genes with antisense transcripts have a much lower stability than all genes or genes with antisense that are not TF. Now this is a bit unfair comparison as the TF with AS should be compared to TF without AS and not to all genes. As for now, data suggest that it is NOT the AS per see that results in lower stability of these genes.

We think there might be misunderstanding here, and we thank the reviewer to bring it up so we can explain our reasoning better to avoid confusion. We do not claim that antisense transcription is involved in the mRNA stability of the host gene. To the contrary, our results (Fig. S1, Fig. 2, Fig. S2, Fig. 5, Fig. S5, and Fig. 9) strongly suggest that antisense transcription is not linked to mRNA stability. We have thoroughly edited the text to avoid any speculated notion that antisense transcription might have a general role in determining mRNA stability.

We agree with the reviewer that a comparison with non-PAS TFs is useful to bring clarity to this concern. We have updated Figure 2 and Supplemental Figure 1 with new data including TFs without antisense transcription. The correlation is clear between the length of a gene and the presence of a PAS (Fig. 2A). The data in Figure 2B-D and Supplemental Figure 1 show that TFs in general have a high turnover rate (i.e. independent of antisense transcription). However, genes with a PAS tend to have higher transcription rate, PAS-TFs represent an extreme case of high transcription and rapid turnover at normal growing temperatures. Thus, PAS-TFs show a more dynamic regulation compared to other PAS genes and other TFs. The dynamic regulation of PAS-TFs is also evident during cold exposure (Fig. 5). Our main conclusion is therefore that a nascent RNA detection method (plaNET-seq) complements steady state methods (RNA-seq) that together notice a greater range of cold responsive genes.

2. Somehow linked to the previous point the authors conclude that: "Changes in the active transcription of a gene measured by plaNET-seq have biological relevance, even though the steady-state level of the gene's mRNA is similar to control conditions after cold exposure (Figure 4-5)”. This is a point I raised in my previous version of the review, and I do not feel it has been addressed properly. It could be formally possible that antisense transcription results in high turnover and as a result despite the observed lack of effect on steady-state RNA level changes observed using netSEQ are relevant for the phenotype. However, as described above and acknowledged by the author in the discussion their experimental data do not support the role of antisense in RNA stability control.

Thank you for this comment. Please, see the answer to the previous point where we point out that nothing in our results and previously published reports suggest a general role for antisense transcription in mRNA turnover. Our main conclusion here is that plaNET-seq, in contrast to RNA-seq, can detect those genes with dynamic mRNA regulation, both under normal growing conditions and after exposure to stress. Highly dynamic regulated genes include PAS genes that have high active transcription as exemplified in this manuscript. Furthermore, we have changed the statement identified by the reviewer and similar in the text to get a more balanced manuscript since we detect small but significant increases of mRNA levels of* BBX28 *and* ZAT5 *at certain time points and experiments. Hence, throughout the manuscript we avoid stating “equal” or “no” difference (in terms of mRNA level) and state minor differences instead.

3. Another possibility could simply be that active transcription measured by netSEQ manifests itself at a stable RNA level later on, and this is translated into the phenotype. But this is quite obvious, and in my opinion, is not reflected in the statement: "Changes in the active transcription of a gene measured by plaNET-seq have biological relevance, even though the steady-state level of the gene's mRNA is similar to control conditions after cold exposure (Figure 4-5). " any many similar throughout the manuscript.

We agree with the reviewer that we cannot rule out an increase of mRNA later in the cold response. However, our focus in this manuscript is to identify means and provide a base to study the transient transcriptional response in the context of antisense transcription during cold. In agreement with reviewer, we have downplayed/changed these statements in the text. We would like to stress that the main message is that highly responsive and dynamically regulated genes are challenging to detect with steady state methods because of the built-in constraints of the methods. The statistical significance of the small differences we detect in the steady state levels of* ZAT5 *and* BBX28 *are thus highly dependent on the variation between biological replicates, stress treatment, and circadian regulation (as pointed out by reviewer 3, see Supplemental Figure S3A-B). In contrast, a nascent detection method increases the probability of finding significance in transcriptional activity over a gene body since it is independent of the high mRNA turnover for certain mRNAs.

4. Finally, the manuscript contains data that suggest an activating role of antisense. Here the data looks quite convincing but in my view is preliminary. The model assumes that AS is downregulated by cold and as a result, sense transcription is induced. That could be true but requires analysis of the separated sense and antisense promoter-reporter fusions for conclusive evidence.

Our data from the CRISPR lines (Fig. 9) and LUC-fusion constructs (Fig. S4) cohesively suggest an activating role for antisense transcription. LUC-fusion constructs with and without antisense transcription disentangled the cis regulatory aspect. The low expression level and high turnover for the antisense transcripts studied here clearly supports a cis role. The experiment suggested by the reviewer would determine trans roles for antisense transcripts which is not a focus of this manuscript. However, we agree with the reviewer that these results are preliminary but quite convincing, and we have therefore been very careful not to state this as a conclusion for a general

role in the results and commented on it in the discussion to get a balanced manuscript. We do conclude that a proper regulation of* ZAT5 *and* BBX28 *require antisense transcription (see Fig. S4, Fig. 9) but the exact mechanism(s), of how this regulation is achieved, we believe is outside the scope of this manuscript but an obvious future avenue of research.

Reviewer #3 (Comments for the Author):

In this resubmitted manuscript, Meena and colleagues have addressed a number of concerns raised during the first round of review. The updated manuscript has substantially improved documentation of data sources, code, and analysis methods. They have also performed additional experiments to better characterize the CRISPR mutant lines and the effect of the 3' region of ZAT5 on protein abundance. Most of my initial concerns have been adequately addressed, and I think the overall ideas presented in the manuscript are worthwhile. However, I still have questions about the relevance of antisense transcription in the two selected genes, ZAT5 and BBX28.

In both the RNA-seq and plaNET-seq, differential expression is calculated vs. the 22°C 0hr timepoint, meaning DEGs are a product of both changing temperature and changing time. Both ZAT5 and BBX28 are reported as circadian-regulated genes (see e.g. Romanowski 2020 Table S3, https://doi.org/10.1111/tpj.14776), so their expression is expected to change over a 12-hour time course regardless of temperature. Have the authors performed any experiments that disentangle temperature effects and circadian effects? For example, comparing to "mock" 3hr and 12hr plants kept at 22°C?

This is a great point and we have included new data in Supplemental Figure S3A-B. The new data show that* BBX28 *had a small but significant increase in cold conditions after 3h and 12h.* ZAT5 *showed a significant increase after 3h but not after 12h. We have also added the reference to the text.

Some new results in Figure 9 conflict with other earlier sections in the paper: [Line 241] states that "the steady-state level of ZAT5 was not significantly increased" in contrast with plaNET-seq results, and this is explained by an increase in ZAT5 turnover in cold (Fig5D). But the new measurements in Fig9B show a larger fold-increase of steady-state ZAT5 than nascent ZAT5 after 3 hours at 4°C. How can this observation be reconciled with the other results? [Line 237] The question "Can mRNAs with equal steady state levels during stress have an important biological role in the stress adaptation for the plant?" is not answered by the examples of ZAT5 or BBX28, because their steady state levels do not stay unchanged.

We thank the reviewer for this comment. Together with the previous comment, it allows us to reconcile our results into an enhanced manuscript. The time series in Figure 5D did not find a significant increase of* ZAT5 *after 2h and 4h of cold. In Figure 9, we have used 3h of cold treatment and we see a small but significant increase (also reflected in the new Supplemental Figure 3B). These results highlight the small differences in mRNA levels at different time points which lead to significant results in some experiments but not in others, largely depending on the variation between biological replicates.

We agree with the reviewer that the question posed on line 237 was incorrectly formulated. We have changed this to "Can mRNAs with small differences in steady state levels during stress have an important biological role in the stress adaptation for the plant?" as this better captures the main message of the manuscript. Furthermore, we have toned down the rest of the text concerning similar statements as the question on line 237 for a more balanced manuscript.

Line 147-150: Examining transcription factors as a separate group is an interesting idea, but Sorenson et al. observed that TFs generally have a higher turnover rate than non-TF genes. Whether PAS transcription specifically is associated with a higher turnover rate of transcription factor RNA would be better demonstrated by comparing "PAS TFs" to "non-PAS TFs", instead of to "PAS non-TFs"

Thank you for this comment and suggestion. See answer to point 1 from reviewer 2.

Minor points:

The most well-studied cold responsive antisense RNA in Arabidopsis is COOLAIR (AT5G01675). It matches the definition of PAS gene defined here, so I am surprised not to see FLC (AT5G10140) listed in the set of PAS host genes in Supplemental Dataset 1. Could the authors clarify why FLC/COOLAIR was not identified in this study?

A good point that needs clarification. The list of PAS genes used in this manuscript are genes without an annotated antisense transcript prior to the plaNET-seq analysis (described in Kindgren et al, 2020). Thus, genes like **FLC** ***and its antisense transcript*** **COOLAIR** ***and a few other well described and characterized antisense transcripts are not part of the list. We have clarified this in the text to avoid any confusion.***

Throughout the manuscript the authors refer to "genes hosting antisense transcripts"; because these are nascent polII products that aren't detected in the steady state pool, would the more accurate phrase be "genes hosting antisense transcription"?

Agreed. This has been changed in the manuscript.

Line 50 could be reworded to "What importance does PAS transcription have in the cold response?"

Fixed.

Dear Dr. Peter Kindgren:

We are pleased to inform you that your paper entitled "Antisense transcription from stress responsive transcription factors fine tunes the cold response in Arabidopsis thaliana: insights from BBX28 and ZAT5 characterization." has been accepted for publication in The Plant Cell, pending a final editorial review by a science editor. At this stage, your manuscript will be evaluated by a science editor with respect to its presentation of scientific content, compliance with journal policies, and presentation for a broad readership. The Plant Cell has contracted with Plant Editors (planteditors.com) to provide this service to our authors, and you will soon receive additional information on this process.

Please note that each author needs to link their ORCID identifier to their account in the system before your manuscript can be published. If any authors do not have an ORCID linked to their account, they will receive a message with a link to complete this task. Please ensure that ALL of your coauthors have completed this task as soon as possible.

ASPB offers an OPEN option that allows authors to have their online articles available for free to all users immediately upon publication. For more information about the ASPB OPEN option, refer to the Final Submission Checklist Form.

The Plant Cell and The Arabidopsis Information Resource (TAIR) are collaborating to collect functional annotation data about Arabidopsis genes from authors. This includes information about the gene's molecular function (e.g., kinase activity, ATP synthetase activity), the biological process/es it is involved in (e.g., endosperm development, threonine biosynthesis), its subcellular location (e.g., nucleus, ER), anatomical or developmental expression pattern (e.g., leaf, ovule, flower stage 10, seedling stage), or its partner in a protein-protein interaction (e.g., AT1G01010 interacts with AT1G01020).

If your paper contains results falling into one or more of these categories for Arabidopsis genes, we request that you now submit these data for inclusion in TAIR by filling in the form provided at the following URL: https://www.arabidopsis.org/doc/submit/functional_annotation/123. If you need further clarification on what types of data can be submitted please contact curator@arabidopsis.org.

Finally, we encourage your submission of artwork for the journal cover. Monthly issues will have an online cover image and selected covers will be used for posters, other promotional items, and "wallpaper" for mobile devices. For more information, refer to Cover Submission in the Instructions for Authors https://tpc.msubmit.net/cgi-bin/main.plex?form_type=display_auth_instructions.

We look forward to seeing your paper published.

Sincerely,

The Plant Cell Editorial Board

=====
IMPORTANT REMINDER: PEER REVIEW REPORTS
=====

If you opted to publish a peer review report along with your article during the original submission process, it will be prepared by the editorial staff and publicly posted with your manuscript, inside the zip file that contains any other supplemental material. As a reminder, the peer review report is a public record of all comments from editors and reviewers, as well as your prior responses, as you received them in the decision letters for each draft of your manuscript. If you agreed to publish this report and have changed your mind, or are not sure if you selected this option, please contact the editorial office as soon as possible before signing the license agreement from our publisher.

=====

----FOR ASPB OFFICE USE ONLY (DO NOT EDIT)----
MSID: 37662
Corresponding Author: Dr. Kindgren
